# Analysis of Clinical Traits Associated With Cardiovascular Health, Genomic Profiles, and Neuroimaging Markers of Brain Health in Adults Without Stroke or Dementia

**DOI:** 10.1001/jamanetworkopen.2022.15328

**Published:** 2022-05-27

**Authors:** Julián N. Acosta, Cameron P. Both, Cyprien Rivier, Natalia Szejko, Audrey C. Leasure, Thomas M. Gill, Seyedmehdi Payabvash, Kevin N. Sheth, Guido J. Falcone

**Affiliations:** 1 Department of Neurology, Yale School of Medicine, New Haven, Connecticut; 2 Department of Internal Medicine, Yale School of Medicine, New Haven, Connecticut; 3 Department of Radiology, Yale School of Medicine, New Haven, Connecticut

## Abstract

**Importance:**

The American Heart Association (AHA) Life’s Simple 7 (LS7) score captures 7 biological and lifestyle factors associated with promoting cardiovascular health.

**Objectives:**

To test whether healthier LS7 profiles are associated with significant brain health benefits in persons without stroke or dementia, and to evaluate whether genomic information can recapitulate the observed LS7.

**Design, Setting, and Participants:**

This genetic association study was a nested neuroimaging study within the UK Biobank, a large population-based cohort study in the United Kingdom. Between March 2006 and October 2010, the UK Biobank enrolled 502 480 community-dwelling persons aged 40 to 69 years at recruitment. This study focused on a subset of 35 914 participants without stroke or dementia who completed research brain magnetic resonance imaging (MRI) and had available genome-wide data. All analyses were conducted between March 2021 and March 2022.

**Exposures:**

The LS7 (blood pressure, low-density lipoprotein cholesterol, hemoglobin A_1c_, smoking, exercise, diet, and body mass index) profiles were ascertained clinically and genomically. Independent genetic variants known to influence each of the traits included in the LS7 were assessed. The total LS7 score ranges from 0 (worst) to 14 (best) and was categorized as poor (≤4), average (>4 to 9) and optimal (>9).

**Main Outcomes and Measures:**

The outcomes of interest were 2 neuroimaging markers of brain health: white matter hyperintensity (WMH) volume and brain volume (BV).

**Results:**

The final analytical sample included 35 914 participants (mean [SD] age 64.1 [7.6] years; 18 830 [52.4%] women). For WMH, compared with persons with poor observed LS7 profiles, those with average profiles had 16% (β = −0.18; SE, 0.03; *P* < .001) lower mean volume and those with optimal profiles had 39% (β = −0.39; SE, 0.03; *P* < .001) lower mean volume. Similar results were obtained using the genomic LS7 for WMH (average LS7 profile: β = −0.06; SE, 0.014; *P* < .001; optimal LS7 profile: β = −0.08; SE, 0.018; *P* < .001). For BV, compared with persons with poor observed LS7 profiles, those with average LS7 profiles had 0.55% (β = 0.09; SE, 0.02; *P* < .001) higher volume, and those with optimal LS7 profiles had 1.9% (β = 0.14; SE, 0.02; *P* < .001) higher volume. The genomic LS7 profiles were not associated with BV.

**Conclusions and Relevance:**

These findings suggest that healthier LS7 profiles were associated with better profiles of 2 neuroimaging markers of brain health in persons without stroke or dementia, indicating that cardiovascular health optimization was associated with improved brain health in asymptomatic persons. Genomic information appropriately recapitulated 1 of these associations, confirming the feasibility of modeling the LS7 genomically and pointing to an important role of genetic predisposition in the observed association among cardiometabolic and lifestyle factors and brain health.

## Key Points

**Question:**

Are observed and genomic American Heart Association Life’s Simple 7 scores associated with brain imaging markers of brain health in persons without stroke or dementia?

**Findings:**

This genetic association study among 35 914 participants from the UK Biobank study found that better observed Life’s Simple 7 profiles were associated with lower white matter hyperintensity volume and higher brain volume and better genomic Life’s Simple 7 profiles were associated with lower white matter hyperintensity volumes.

**Meaning:**

These findings suggest that cardiovascular health optimization, as measured by Life’s Simply 7 score, was associated with improved brain health in asymptomatic persons.

## Teaser

This genetic association study assesses the associations of observed and genetic cardiovascular health profiles with brain health among asymptomatic adults.

## Introduction

The American Heart Association Life’s Simple 7 (LS7) score captures 7 biological and lifestyle traits associated with a person’s overall cardiovascular health status.^1^ Higher LS7 scores, indicating better cardiovascular health, are associated with lower risk of acute cardiovascular events^2^ and dementia.^3^ Beyond these clinical end points, a few recent studies have evaluated whether better cardiovascular health is associated with better brain health, as measured by neuroimaging markers.^4^ In this study, we hypothesized that better LS7 profiles are associated with significant brain health benefits, as evaluated using 2 neuroimaging markers, in persons without stroke or dementia. Because the biological and lifestyle factors contained in the LS7 are highly heritable, we also hypothesize that the genetic predisposition to these traits can be expressed as the genomic LS7.

## Methods

This genetic association study was approved by the North-West Multi-center Research Ethics Committee. All participants provided electronic informed consent. This study is reported following the Strengthening the Reporting of Genetic Association Studies (STREGA) reporting guidelines.

### Study Design

The UK Biobank is a large population-based cohort study that enrolled 502 480 community-dwelling persons in the United Kingdom aged 40 to 69 years at recruitment between March 2006 and October 2010.^5^ We conducted a nested study within the UK Biobank focusing on participants who underwent dedicated research brain magnetic resonance imaging (MRI).^5^ We included participants with available genetic and neuroimaging data who did not have a history of stroke or dementia at the time of the neuroimaging assessment. Stroke and dementia were ascertained via self-reported data obtained during the baseline visit and previously validated *International Statistical Classification of Diseases and Related Health Problems, Tenth Revision* (*ICD-10*) codes^6,7^ abstracted from electronic health records of hospital admissions and primary care visits that took place between enrollment and the neuroimaging assessment. All analyses were conducted between March 2021 and March 2022.

### Observed and Genomic Life’s Simple 7

Details about the protocol followed to obtain self-reported information, electronic health records, blood pressure measurements, and laboratory data are available elsewhere.^5^ The observed LS7 scores were ascertained using values for blood pressure, low-density lipoprotein cholesterol, glycemic status (hemoglobin A_1c_), smoking, exercise, diet, and body mass index at the time of enrollment (eTable 1 in the Supplement).^8,9^ Each component is codified as either 0 (poor), 1 (average), or 2 (optimal).^3^ Clinical, blood pressure, and biometric data were obtained during the baseline interview. Laboratory data were obtained from blood obtained during the baseline visit. A detailed description of DNA collection, genotyping, quality control, and imputation procedures is available elsewhere.^5^ The genomic LS7 scores were calculated using 7 polygenic risk scores, 1 for each trait captured by the LS7. These polygenic risk scores were calculated using a previously curated and validated list of independent (*r*^2^ < 0.001) genetic risk variants known to influence the 7 components of the LS7 (eTable 2 in the Supplement).^3^ Each polygenic risk score was calculated as the sum of the products of the allele count at each locus multiplied by the effect of that allele on the corresponding trait.^10^ To emulate the categorization into 3 levels, we divided each polygenic risk score into tertiles and assigned participants a value of 0 (higher polygenic risk), 1 (average polygenic risk), and 2 (lower polygenic risk) (eFigure 1 in the Supplement). The total observed and genomic LS7 scores are calculated by summing up all the components, with a final score that ranges from 0 (worst) to 14 (best). This final score was further categorized as poor (≤4), average (>4 to 9), and optimal (>9).

### Outcome of Interest

Details about the MRI neuroimaging research protocol used by the UK Biobank are available elsewhere.^11^ Briefly, a subset of the UK Biobank participants consented to the neuroimaging study. These participants underwent a dedicated research brain MRI imaging using a Siemens Skyra 3T. Brain volume (BV) and white matter hyperintensity (WMH) volume were calculated by the UK Biobank research team using previously validated tools.^11,12^ Our 2 main outcomes of interest were the WMH volume and BV normalized by head size. WMH volume was natural log–transformed to approximate normality. Both natural log–transformed WMH volume and BV were standardized by subtracting the mean and dividing by the SD.

### Statistical Analysis

Because the genetic variants used in the study were discovered in studies including persons of European ancestry and that population stratification can lead to false positive associations, we restricted our main analysis to participants from genetically ascertained European ancestry. We tested for associations between the observed and genomic LS7 profiles and WMH volume and BV using multivariable linear regression adjusting for age and sex. For genomic LS7, we also adjusted for the first 4 genetic principal components. We conducted the 9 analyses in addition to our primary analysis. First, we evaluated participants of all ancestral groups. Second, we developed and tested 1 subscore for lifestyle components (ie, smoking status, body mass index, physical activity, and diet) and a second for the biological components (ie, blood pressure, cholesterol levels, and glycemic status). Third, we evaluated the association between each of the 7 traits and our outcomes of interest. Fourth, we tested the association between the continuous LS7 scores and our outcomes of interest. Fifth, we developed a polygenic score, including genetic variants for all these traits, and tested its association as a continuous variable with our outcomes of interest. Sixth, we tested the association between the LS7 profiles and 7 cognitive measurements provided by the UK Biobank. Seventh, we conducted stratified analyses and interaction analysis by sex. Eighth, we evaluated the correlation between the LS7 and both lifestyle and biological components and the overlap in single-nucleotide variations (SNVs; formerly single-nucleotide polymorphisms) shared by traits. Ninth, we tested the proportion of variance explained by models, including the observed and genomic LS7 scores and components.

For our primary analysis, we used a Bonferroni corrected 2-tailed *P* value of .05 / 4 = .0125 to determine statistical significance. Polygenic risk scoring was conducted using PRSice-2 (PRSice). Analyses were conducted using R statistical software version 3.6.3 (R Project for Statistical Computing).

## Results

The final analytical sample included 35 914 participants (mean [SD] age, 64.1 [7.6] years, 18 830 [52.4%] women) (Table 1). The mean (SD) time from enrollment to the neuroimaging assessment was 8.93 (1.8) years. The flowchart of included participants is available in eFigure 2 in the Supplement.

**Table 1.** Baseline Characteristics of the Study Population

Characteristic	No. (%)		
	Overall (N = 35 914)	Women (n = 18 830)^a^	Men (n = 17 084)^a^
Age, mean (SD), y	64.13 (7.64)	63.46 (7.49)	64.88 (7.74)
Hypertension	6723 (18.7)	2837 (15.1)	3886 (22.7)
Hyperlipidemia	3137 (8.7)	1092 (5.8)	2045 (12.0)
Diabetes	842 (2.3)	283 (1.5)	559 (3.3)
Smoking status			
Never	21 973 (61.3)	12 160 (64.7)	9813 (57.5)
Previous	11 764 (32.8)	5710 (30.4)	6054 (35.5)
Current	2111 (5.9)	922 (4.9)	1189 (7.0)
BMI, mean (SD)	26.55 (4.21)	26.05 (4.54)	27.10 (3.73)
Physically active	19 603 (56.1)	10 133 (55.8)	9470 (56.5)
Healthy diet	1539 (4.3)	981 (5.2)	558 (3.3)
Neuroimaging markers, median (IQR), cm^3^			
WMH volume	3.78 (2.01-7.69)	3.63 (1.96-7.24)	3.97 (2.08-8.16)
Brain volume	1492 (1441-1543)	1504 (1453-1555)	1478 (1431-1527)

Abbreviations: BMI, body mass index (calculated as weight in kilograms divided by height in meters squared); WMH, white matter hyperintensity.

^a^ All differences between men and women participants are statistically significant (all *P* < .001) with the exception of physical activity (*P* = .09).

### WMH Volume

Compared with persons with poor observed LS7 profiles, those with average profiles had 16% (β = −0.18; SE, 0.03; *P* < .001) lower WMH volume, and those with optimal profiles had 39% (β = −0.39; SE, 0.03; *P* < .001) lower WMH volume (Table 2). Similar results were obtained when using the genomic LS7 profiles (average LS7 profile: β = −0.06; SE, 0.014; *P* < .001; optimal LS7 profile: β = −0.08; SE, 0.018; *P* < .001) (Table 2). Sensitivity analyses, including 39 976 participants from all ancestral backgrounds, including those from genetically ascertained African or Asian ancestry, with available neuroimaging and genomic data yielded similar results. In the secondary analyses, the lifestyle and biological components of the LS7 score were each associated with WMH volume for both the observed and genomic LS7 profiles (Table 3). Of note, for the genomic LS7, the association was stronger for the biological vs lifestyle component (Table 3; eTable 3 in the Supplement).

**Table 2.** Multivariable Linear Regression Results Showing Associations Between Observed and Genomic LS7 Scores and Neuroimaging Markers of Brain Health

LS7 profile	White matter hyperintensities volume			Brain volume		
	β (SE)	Absolute change, cc	*P* value	β (SE)	Absolute change, cc	*P* value
Observed						
Poor	0 [Reference]	0 [Reference]	NA	0 [Reference]	0 [Reference]	NA
Average	−0.18 (0.026)	−1.2	<.001	0.09 (0.024)	6.6	<.001
Optimal	−0.39 (0.026)	−2.6	<.001	0.14 (0.025)	10.3	<.001
Genomic						
Poor	0 [Reference]	0 [Reference]	NA	0 [Reference]	0 [Reference]	NA
Average	−0.06 (0.014)	−0.4	<.001	−0.005 (0.01)	−0.4	.69
Optimal	−0.08 (0.018)	−0.5	<.001	−0.02 (0.02)	−1.4	.32

Abbreviations: LS7, Life’s Simple 7; NA, not applicable.

**Table 3.** Multivariable Linear Regression Results Showing Associations Between Lifestyle and Biological Components of Observed and Genomic LS7 and Neuroimaging Markers of Brain Health

LS7 profile (score)	White matter hyperintensities volume		Brain volume	
	β (SE)	*P* value	β (SE)	*P* value
**Lifestyle components** ^a^				
Observed				
Poor (0-2)	0 [Reference]	NA	0 [Reference]	NA
Average (3-5)	−0.23 (0.022)	<.001	0.10 (0.02)	<.001
Optimal (6-8)	−0.34 (0.023)	<.001	0.15 (0.02)	<.001
Genomic				
Poor (0-2)	0 [Reference]	NA	0 [Reference]	NA
Average (3-5)	−0.02 (0.012)	.04	0.004 (0.01)	.71
Optimal (6-8)	−0.03 (0.015)	.05	0.003 (0.01)	.84
**Biological components** ^b^				
Observed				
Poor (0-1)	0 [Reference]	NA	0 [Reference]	NA
Average (2-3)	−0.10 (0.03)	.002	0.01 (0.03)	.64
Optimal (4-6)	−0.24 (0.03)	<.001	0.04 (0.03)	.18
Genomic				
Poor (0-1)	0 [Reference]	NA	0 [Reference]	NA
Average (2-3)	−0.04 (0.014)	.001	−0.005 (0.01)	.69
Optimal (4-6)	−0.08 (0.014)	<.001	−0.01 (0.01)	.42

Abbreviations: LS7, Life’s Simply 7; NA, not applicable.

^a^ Includes smoking status, body mass index, physical activity, and diet. Scores range from 0 to 8.

^b^ Includes blood pressure, cholesterol levels, and glycemic status. Scores range from 0 to 6.

### Brain Volume

Compared with persons with poor observed LS7 profiles, those with average LS7 profiles had 0.55% (β = 0.09; SE, 0.02; *P* < .001) higher BV, and those with optimal LS7 profiles had 1.9% (β = 0.14; SE, 0.02; *P* < .001) higher BV. The genomic LS7 profiles were not associated with BV (Table 2). Sensitivity analyses, including 41 303 participants from all ancestral backgrounds with available neuroimaging and genomic data, yielded similar results. In secondary analyses, only the lifestyle component of the observed LS7 profile was significantly associated with BV (Table 3).

### Additional Analyses

When evaluating the association between both the observed and genomic LS7 scores using the continuous variable instead of the categorized profile variable, we found consistent results. Additionally, when evaluating a continuous polygenic score using genetic variants from all 7 traits, results remained consistent with our primary analysis: each SD of this polygenic score was associated with a decrease of 0.3 cc in WMH volume (β = −0.04; SE, 0.005; *P* < .001), while no significant results were seen for brain volume (β = 0.005; SE, 0.004; *P* = .22). When testing the association between the LS7 profiles and 7 cognitive measurements, we found that better profiles of both the observed and genomic LS7 were associated with higher measures of fluid intelligence and better performance in the symbol digit substitution test (eTable 4 in the Supplement). When analyzing association modification by sex, we found that the association between the observed LS7 profiles and brain volume was only present in men (eTable 5 in the Supplement). All other interaction analyses were not significant. In additional analyses, we found only up to 3 SNVs were shared among traits (eFigure 3 in the Supplement), and that the correlation between the genomic LS7 including all traits and the LS7 including only biological components was moderate (*R* = 0.65; 95% CI, 0.64-0.65; *P* < .001). However, models including all components vs only biological components did not improve significantly (eTable 6 in the Supplement). The addition of the genomic LS7 score to a model containing the observed LS7 score only marginally increased proportion of variance explained (eTable 6 in the Supplement).

## Discussion

In this genetic association study analyzing data from nearly 36 000 persons without stroke or dementia, we found that a better (healthier) observed LS7 profile was associated with lower WMH volume and larger BV. We also found that a better (healthier) genomic LS7 profile was associated with lower WMH volume but not BV. Mounting evidence points to an important link between cardiovascular and brain health.^13^ A previous report from the US-based Northern Manhattan Study^4^ showed a similar association between observed LS7 score and subclinical imaging markers of brain health. Our study supports these findings in a British study population and suggests that the different components of the LS7 score have different associations with brain health: while the lifestyle component was associated with both WMH volume and BV, the biological component was only associated with WMH volume. Additionally, we found that the association between cardiovascular health and brain volume may differ by sex, with our results only being significant among men in stratified analyses.

Our study provides important new findings on this topic by evaluating the association between cardiovascular and brain health from a genetic perspective. We show that it is possible to calculate a genomic version of the LS7 score using the numerous genetic variants that are known to influence each of its 7 traits. The validity of this approach is demonstrated by the significant association between the genomic LS7 profiles and WMH volume. These findings suggest that an individual’s genetic predisposition constitutes an important determinant of neuroimaging biomarkers of brain health. These biomarkers have been shown to be associated, in turn, with clinical end points, such as cognitive performance, stroke, and dementia.^14,15,16,17^ Along these lines, we found that better LS7 profiles might be associated with improved cognitive function. Finally, because genetic information from SNVs is present since birth and remains mostly unchanged throughout life, our findings lay the foundation for future research focused on evaluating whether the use of the genomic LS7 could lead to the ultra-early identification of individuals with high risk who could benefit from tailored diagnostic or therapeutic interventions.^18^ Along these lines, a few studies have shown that communicating polygenic risk is associated with positive changes in health behavior.^19,20^

### Limitations

Our study has several limitations. First, some lifestyle traits, such as diet and physical activity, do not have well-established genetic instruments, limiting our results for these traits. Furthermore, the categorization of the LS7 scores into profiles may remove information, adding noise to our analyses. However, when testing these associations using continuous variables, results remained consistent. Additionally, the variables included in the observed LS7 score were only evaluated at a single point in time (at recruitment), which further limits our analyses. In addition, we did not find an association between the genomic LS7 profiles and BV. These null results could be due to the inevitable decrease in statistical power produced by working with genetic proxies of cardiometabolic traits instead of the observed traits. Alternatively, these null results could indicate that the association between the observed the LS7 profiles and BV identified by this and prior observational studies may not be causal.

## Conclusions

This genetic association study found that better cardiovascular health profiles, as expressed by the LS7, were associated with better neuroimaging-defined brain health in persons without stroke or dementia. These findings suggest that it may be possible to use genetic information from variants known to influence the 7 cardiometabolic and lifestyle traits contained in the LS7 to calculate the genomic LS7 score.

## Notes

Supplement. **eTable 1.** Definition of Life’s Simple 7 Score Components in the UK Biobank **eTable 2.** Genetic Variants Used to Develop the Genomic Life’s Simple 7 Score **eTable 3.** Results for Individual Life’s Simple 7 Components as Factors Associated With Neuroimaging-Related Brain Health Metrics **eTable 4.** Association Between Life’s Simple 7 Scores and Cognitive Measurements in the UK Biobank **eTable 5.** Stratified Analyses by Sex **eTable 6.** Comparison of Performance of Different Models for Each Trait **eFigure 1.** Development of the Genomic Life’s Simple 7 Score **eFigure 2.** Flowchart of UK Biobank Participants Included in the Primary Analysis **eFigure 3.** Single Nucleotide Variants Included in the Analysis and Overlap Among Traits
