# Supplementary material for: Analysis of Clinical Traits Associated With Cardiovascular Health, Genomic Profiles, and Neuroimaging Markers of Brain Health in Adults Without Stroke or Dementia
Source: JAMA Netw Open. 2022 May 27;5(5):e2215328. doi: 10.1001/jamanetworkopen.2022.15328 (PMC9142873; doi:10.1001/jamanetworkopen.2022.15328)
Supplement: Supplement. — eTable 1. Definition of Life’s Simple 7 Score Components in the UK Biobank eTable 2. Genetic Variants Used to Develop the Genomic Life’s Simple 7 Score eTable 3. Results for Individual Life’s Simple 7 Components as Factors Associated With Neuroimaging-Related Brain Health Metrics eTable 4. Association Between Life’s Simple 7 Scores and Cognitive Measurements in the UK Biobank eTable 5. Stratified Analyses by Sex eTable 6. Comparison of Performance of Different Models for Each Trait eFigure 1. Development of the Genomic Life’s Simple 7 Score eFigure 2. Flowchart of UK Biobank Participants Included in the Primary Analysis eFigure 3. Single Nucleotide Variants Included in the Analysis and Overlap Among Traits [file jamanetwopen-e2215328-s001.pdf]

Supplemental Online Content

Acosta JN, Both CP, Rivier C, et al. Analysis of clinical traits associated with cardiovascular health, genomic profiles, and neuroimaging markers of brain health in adults without stroke or dementia. *JAMA Netw Open*. 2022;5(5):e2215328. doi:10.1001/jamanetworkopen.2022.15328

eTable 1. Definition of Life's Simple 7 Score Components in the UK Biobank
eTable 2. Genetic Variants Used to Develop the Genomic Life's Simple 7 Score
eTable 3. Results for Individual Life's Simple 7 Components as Factors Associated With Neuroimaging-Related Brain Health Metrics
eTable 4. Association Between Life's Simple 7 Scores and Cognitive Measurements in the UK Biobank
eTable 5. Stratified Analyses by Sex
eTable 6. Comparison of Performance of Different Models for Each Trait
eFigure 1. Development of the Genomic Life's Simple 7 Score
eFigure 2. Flowchart of UK Biobank Participants Included in the Primary Analysis
eFigure 3. Single Nucleotide Variants Included in the Analysis and Overlap Among Traits

This supplemental material has been provided by the authors to give readers additional information about their work.

© 2022 Acosta JN et al. *JAMA Network Open.*

eTable 1. Definition of Life's Simple 7 Score Components in the UK Biobank

Life's Simple 7 score	Lifestyle components				Biological components		
	**Smoking status**	**BMI**	**Physical activity**	**Diet***	**Blood pressure**	**Cholesterol levels**	**Glycemic status**
**Optimal (score = 2)**	Never smoked	<25 kg/m2	>4 days/week of moderate/vigorous physical activity	>7	SBP <120 mm Hg and DBP <80 mm Hg untreated	LDL-C <130 mg/dl	HbA1c <5.7%
**Average (score = 1)**	Former smoker	25-29.9 kg/m2	2 ≤4 days/week of moderate/vigorous physical activity	4-7	SBP 120-139 or DBP 80-89 mm Hg OR SBP <120 mm Hg and DBP <80 mm Hg treated	LDL-C 130-159 mg/dl OR LDL-C <130 mg/dl treated	HbA1c 5.7-6.4% OR HbA1c <5.7% treated
**Poor (score = 0)**	Current smoker	≥30 kg/m2	No moderate/ vigorous physical activity	<4	SBP ≥140 mm Hg or DBP ≥90 mm Hg	LDL-C ≥ 160 mg/dl	HbA1c ≥ 6.4%

Adapted from Malik et al.[3] *Healthy diet score according to Mozaffarian[9] and Said et al.[8]; higher scores indicate adherence to a healthier diet for prevention of cardiovascular disease. Abbreviations: BMI = body mass index; SBP = systolic blood pressure; DBP = diastolic blood pressure; LDL-C = low-density lipoprotein cholesterol; HbA1c = glycated hemoglobin.

© 2022 Acosta JN et al. *JAMA Network Open.*

eTable 2. Genetic Variants Used to Develop the Genomic Life's Simple 7 Score

SNP	Effect Allele	Beta	SE	Trait
rs1000096	T	-0.0144	0.0018	bmi
rs1000940	A	-0.0154	0.0018	bmi
rs1003081	T	0.0121	0.0016	bmi
rs10035289	A	0.0085	0.0017	bmi
rs10037047	A	-0.0056	0.0023	bmi
rs1006353	A	0.0126	0.0019	bmi
rs10066835	T	0.0428	0.0074	bmi
rs10083803	T	-0.0127	0.0019	bmi
rs10110727	A	0.0135	0.002	bmi
rs10118701	A	-0.0163	0.0018	bmi
rs10131890	A	-0.0222	0.0039	bmi
rs10132280	A	-0.0223	0.0018	bmi
rs1014194	A	0.0102	0.0018	bmi
rs10146527	T	0.0137	0.0017	bmi
rs1015362	T	-0.0038	0.0019	bmi
rs1015363	A	-0.0024	0.0017	bmi
rs10182181	A	-0.0325	0.0016	bmi
rs10190332	T	0.0037	0.0019	bmi
rs10203277	A	0.0044	0.0025	bmi
rs1020548	A	-0.0132	0.0023	bmi
rs10211055	T	-0.0157	0.0018	bmi
rs10268050	T	-0.0113	0.002	bmi
rs10269783	A	0.0133	0.0017	bmi
rs1035010	T	0.0139	0.002	bmi
rs1037587	T	0.0106	0.0017	bmi
rs1038088	T	-0.0117	0.0016	bmi
rs10408013	T	0.0111	0.0018	bmi
rs1040881	T	0.0106	0.0018	bmi
rs10438964	T	-0.0127	0.0019	bmi
rs1045411	T	-0.0148	0.0019	bmi
rs10459012	A	0.0084	0.002	bmi
rs10460960	A	0.0197	0.0025	bmi
rs1048365	T	0.0136	0.0025	bmi
rs1048932	A	-0.016	0.0017	bmi
rs10499276	T	0.0172	0.0025	bmi
rs10500548	T	0.0188	0.0036	bmi
rs10510321	T	0.0114	0.0021	bmi
rs10511093	T	-0.0228	0.0034	bmi
rs10515050	T	-0.0077	0.0017	bmi
rs1057452	A	0.0173	0.0024	bmi
rs10733051	A	0.0097	0.0016	bmi
rs10744146	A	-0.0122	0.0017	bmi
rs10745785	T	-0.0111	0.0018	bmi
rs10754210	A	-0.0123	0.0018	bmi
rs1075901	T	-0.0121	0.0016	bmi
rs10769165	T	-0.0067	0.0016	bmi
rs10773049	T	-0.0116	0.0017	bmi
rs10779751	A	0.0139	0.0018	bmi
rs10797115	T	0.0124	0.0017	bmi
rs10818810	A	0.0126	0.0017	bmi
rs10818938	A	0.0114	0.0017	bmi
rs10830452	A	-0.0111	0.0018	bmi
rs10838122	T	-0.0105	0.0017	bmi
rs10838852	T	0.0053	0.0017	bmi
rs10839472	T	0.0044	0.002	bmi
rs10840606	A	-0.0164	0.0024	bmi
rs10840674	A	-0.0089	0.0018	bmi
rs10850031	T	-0.0075	0.0018	bmi
rs10865858	T	0.0092	0.0017	bmi
rs10867256	T	-0.0118	0.0017	bmi
rs10876418	T	-0.0123	0.0019	bmi
rs10878946	T	-0.0141	0.0019	bmi
rs10883553	A	0.0119	0.0018	bmi

© 2022 Acosta JN et al. *JAMA Network Open.*

SNP	Effect Allele	Beta	SE	Trait
rs10883759	A	-0.0122	0.0018	bmi
rs10886017	A	0.0152	0.0019	bmi
rs10915840	A	-0.0118	0.0019	bmi
rs10920678	A	0.0155	0.0016	bmi
rs10923724	T	-0.0118	0.0016	bmi
rs10929925	A	-0.0143	0.0016	bmi
rs10930641	A	-0.0137	0.0017	bmi
rs10935143	A	-0.0109	0.0017	bmi
rs10938397	A	-0.0324	0.0016	bmi
rs10953620	A	-0.0101	0.0017	bmi
rs10955841	A	0.0096	0.0018	bmi
rs10962549	T	0.0198	0.0023	bmi
rs10971712	T	-0.0197	0.0027	bmi
rs10989568	A	0.0107	0.0017	bmi
rs11030385	A	-0.0084	0.0018	bmi
rs11039014	A	4.00E-04	0.0019	bmi
rs11066301	A	0.0114	0.0016	bmi
rs1106908	A	-0.0158	0.0016	bmi
rs11074446	T	0.0225	0.0024	bmi
rs11075489	T	-0.0111	0.0017	bmi
rs11079849	T	-0.0188	0.0019	bmi
rs11115176	T	0.0121	0.0019	bmi
rs11118308	A	0.0101	0.0016	bmi
rs11128760	A	0.011	0.0017	bmi
rs11150911	A	0.0133	0.0018	bmi
rs11172702	A	0.0144	0.0033	bmi
rs11190661	T	0.001	0.0018	bmi
rs11231548	A	-0.0278	0.006	bmi
rs11250076	A	0.0184	0.0018	bmi
rs11251352	A	-0.0109	0.0018	bmi
rs11259933	A	-0.0066	0.0017	bmi
rs11496125	T	0.0169	0.0017	bmi
rs11577179	A	-0.0108	0.0017	bmi
rs1158103	A	-0.009	0.0017	bmi
rs11583122	T	0.0182	0.003	bmi
rs11590474	T	-0.005	0.0021	bmi
rs11600990	T	-0.0178	0.0023	bmi
rs11611246	T	0.024	0.002	bmi
rs11611496	A	-0.0169	0.002	bmi
rs11635675	T	0.0124	0.0018	bmi
rs11642001	A	-0.0137	0.0021	bmi
rs11668301	A	0.021	0.0024	bmi
rs11672660	T	-0.034	0.0021	bmi
rs11753081	T	0.0138	0.0021	bmi
rs11773362	T	-0.0111	0.0018	bmi
rs1178060	A	0.0193	0.0023	bmi
rs11781222	T	0.0158	0.0024	bmi
rs11781699	T	-0.0132	0.0021	bmi
rs11783247	T	-0.0169	0.0016	bmi
rs11790280	T	-0.0103	0.0018	bmi
rs11855853	T	-0.0145	0.002	bmi
rs11866815	T	-0.0156	0.0019	bmi
rs11889536	A	0.0189	0.0024	bmi
rs11915371	A	-0.0149	0.0021	bmi
rs11929028	T	0.0174	0.0033	bmi
rs11945861	A	-0.0148	0.002	bmi
rs11971041	A	-0.021	0.0031	bmi
rs11987383	A	-0.0147	0.0026	bmi
rs12033257	A	0.0146	0.0018	bmi
rs12035349	A	-0.0188	0.0026	bmi
rs12039524	A	0.0011	0.0023	bmi
rs12041258	T	0.0146	0.002	bmi
rs12042908	A	0.0184	0.0016	bmi
rs12042959	A	0.0144	0.0024	bmi
rs12044597	A	-0.0143	0.0016	bmi

© 2022 Acosta JN et al. *JAMA Network Open.*

SNP	Effect Allele	Beta	SE	Trait
rs1205106	A	-0.0084	0.0017	bmi
rs12098284	T	0.0178	0.0026	bmi
rs12147845	T	0.0199	0.0027	bmi
rs12148386	T	-0.0049	0.0017	bmi
rs1218822	A	0.0168	0.0017	bmi
rs12189178	T	0.0364	0.0046	bmi
rs12207241	A	-0.0076	0.0018	bmi
rs12215331	T	0.0177	0.002	bmi
rs1229057	T	0.017	0.0027	bmi
rs12306932	T	-8.00E-04	0.0016	bmi
rs12327272	A	0.0053	0.0027	bmi
rs12364470	T	-0.0178	0.0022	bmi
rs12369009	T	-0.0019	0.002	bmi
rs12411886	A	0.0271	0.003	bmi
rs12417072	A	-0.0151	0.0027	bmi
rs1241986	A	-0.0139	0.0024	bmi
rs12420725	A	-0.0226	0.0037	bmi
rs12429545	A	0.0316	0.0025	bmi
rs12439798	T	0.0125	0.0017	bmi
rs12443621	A	-0.0096	0.0017	bmi
rs12446632	A	-0.0352	0.0024	bmi
rs12448257	A	0.0184	0.002	bmi
rs12448738	A	-0.0168	0.0025	bmi
rs12453418	A	-0.0096	0.0018	bmi
rs12480713	T	0.007	0.0018	bmi
rs12488237	T	-0.0235	0.0036	bmi
rs12514473	T	0.0167	0.002	bmi
rs12564992	A	-0.0196	0.0026	bmi
rs12574668	A	0.0132	0.0023	bmi
rs12587412	T	0.0146	0.0017	bmi
rs12593036	A	0.0154	0.0019	bmi
rs12595158	T	-0.0394	0.0054	bmi
rs12595749	A	0.0141	0.0017	bmi
rs12602912	T	0.0176	0.0021	bmi
rs12609744	T	-0.0126	0.0019	bmi
rs12615778	A	0.0104	0.0019	bmi
rs12625413	T	-0.0079	0.0018	bmi
rs12637576	T	0.0024	0.0018	bmi
rs12638746	A	-0.0092	0.0018	bmi
rs12659802	A	0.0089	0.0018	bmi
rs12666574	A	0.0097	0.0018	bmi
rs1266922	A	7.00E-04	0.0018	bmi
rs12680842	A	0.0133	0.0018	bmi
rs12682565	A	0.0232	0.0046	bmi
rs12694021	A	0.0106	0.0017	bmi
rs12713433	T	0.0088	0.0021	bmi
rs12731372	T	0.0116	0.0019	bmi
rs12885454	A	-0.0185	0.0017	bmi
rs12888545	A	-0.0136	0.002	bmi
rs12920590	T	-0.0029	0.0019	bmi
rs12939549	A	0.018	0.0016	bmi
rs1296328	A	0.0179	0.0018	bmi
rs12964689	A	0.0203	0.0017	bmi
rs13021737	A	-0.0574	0.0021	bmi
rs13041173	A	-0.0107	0.0018	bmi
rs13045538	T	-0.0088	0.0052	bmi
rs13072095	T	0.0095	0.0017	bmi
rs13085472	T	0.0098	0.0018	bmi
rs13095652	T	-0.0085	0.0018	bmi
rs13107325	T	0.047	0.0032	bmi
rs13110266	A	-0.0117	0.0017	bmi
rs13163306	A	-0.0094	0.0017	bmi
rs13174863	A	-0.0192	0.0023	bmi
rs13175892	T	-0.0215	0.0054	bmi
rs13191362	A	0.0236	0.0025	bmi

© 2022 Acosta JN et al. *JAMA Network Open.*

SNP	Effect Allele	Beta	SE	Trait
rs13201877	A	-0.0152	0.0024	bmi
rs13203153	A	-0.012	0.0022	bmi
rs13203286	T	-0.005	0.0038	bmi
rs13207082	A	-0.0118	0.0028	bmi
rs1320903	A	0.0216	0.0018	bmi
rs13209753	A	-0.0182	0.0036	bmi
rs13227433	T	-0.0156	0.002	bmi
rs13227658	T	-0.0157	0.0017	bmi
rs1323068	A	-0.0114	0.0018	bmi
rs13240600	A	0.0204	0.0024	bmi
rs13247665	T	-0.0139	0.0018	bmi
rs13263601	A	-0.0154	0.0018	bmi
rs13290794	A	-0.0141	0.0018	bmi
rs13292976	T	0.0131	0.0017	bmi
rs13321566	A	-0.0135	0.0023	bmi
rs13329567	T	-0.0293	0.002	bmi
rs13417156	T	-0.0144	0.0017	bmi
rs13432055	T	-0.0117	0.0018	bmi
rs1345148	T	-0.0111	0.0017	bmi
rs1362910	A	0.0121	0.0017	bmi
rs1365466	T	-0.0137	0.0019	bmi
rs1371108	A	0.0119	0.0018	bmi
rs1375561	T	0.0173	0.0017	bmi
rs1394	A	-0.0154	0.0017	bmi
rs1399054	A	-0.0101	0.0022	bmi
rs1403846	T	0.0116	0.0021	bmi
rs1405348	A	-0.0204	0.0017	bmi
rs1423627	T	0.0059	0.0021	bmi
rs1431659	A	0.0196	0.0019	bmi
rs143384	A	-7.00E-04	0.0017	bmi
rs1436343	A	-0.0141	0.0017	bmi
rs1437929	A	0.0135	0.0019	bmi
rs1451533	A	0.0167	0.0019	bmi
rs1452075	T	0.0141	0.0018	bmi
rs1452134	T	-0.0091	0.0017	bmi
rs1454148	T	0.0106	0.0019	bmi
rs1455137	A	-0.0106	0.0017	bmi
rs1473579	A	-0.0026	0.0017	bmi
rs1475774	A	0.0257	0.0058	bmi
rs1477887	A	-0.0138	0.0017	bmi
rs1492767	T	0.0094	0.0016	bmi
rs1498139	A	0.0105	0.002	bmi
rs1503139	A	-0.0059	0.0018	bmi
rs150353	T	-0.0095	0.0018	bmi
rs1512065	A	0.0138	0.0021	bmi
rs1522569	T	0.0164	0.0022	bmi
rs1544459	T	-0.0103	0.0016	bmi
rs1549293	T	-0.0204	0.0017	bmi
rs1564981	A	0.0087	0.0016	bmi
rs159032	T	0.0129	0.002	bmi
rs1593304	A	0.0126	0.0022	bmi
rs1600136	A	-0.0081	0.0021	bmi
rs160401	T	0.0062	0.0019	bmi
rs1608445	A	-0.0104	0.0018	bmi
rs1625427	T	0.013	0.0018	bmi
rs1650586	T	0.0134	0.0028	bmi
rs1657930	A	-0.0123	0.0022	bmi
rs1658820	T	0.0141	0.0021	bmi
rs1668633	T	0.01	0.0017	bmi
rs16823670	A	0.0261	0.0052	bmi
rs16851483	T	0.0369	0.0035	bmi
rs16906845	A	-0.0225	0.0038	bmi
rs16907751	T	-0.0209	0.003	bmi
rs16932761	A	-0.014	0.002	bmi
rs16951319	T	-0.007	0.0025	bmi

© 2022 Acosta JN et al. *JAMA Network Open.*

SNP	Effect Allele	Beta	SE	Trait
rs16965062	T	0.009	0.0017	bmi
rs16966801	A	-0.0156	0.0022	bmi
rs1700137	T	-0.0121	0.0019	bmi
rs17014375	T	-0.0172	0.0025	bmi
rs17035438	A	0.0199	0.003	bmi
rs17056301	T	-0.0118	0.002	bmi
rs17069831	T	-0.0109	0.0019	bmi
rs17080319	T	0.0109	0.0026	bmi
rs17091470	T	0.0015	0.0033	bmi
rs17094222	T	-0.0181	0.002	bmi
rs17096549	A	0.0205	0.0052	bmi
rs17096552	A	-0.0145	0.0025	bmi
rs17105272	T	0.011	0.0019	bmi
rs1712517	T	-0.0028	0.0017	bmi
rs17197116	T	-0.0052	0.003	bmi
rs17199978	A	0.0025	0.0029	bmi
rs17203016	A	-0.015	0.002	bmi
rs17207196	T	-0.0221	0.0018	bmi
rs17238110	A	0.0353	0.005	bmi
rs17285919	T	4.00E-04	0.0026	bmi
rs17302346	T	-0.0201	0.0065	bmi
rs1730859	A	-0.0118	0.0017	bmi
rs17327461	T	0.0125	0.0016	bmi
rs17351791	A	0.0097	0.0018	bmi
rs17391694	T	0.0317	0.0025	bmi
rs17446091	T	-0.0123	0.002	bmi
rs17448682	T	0.0099	0.002	bmi
rs17522122	T	0.0159	0.0017	bmi
rs17525725	A	-0.0083	0.0017	bmi
rs17531363	A	0.0133	0.0019	bmi
rs17538472	T	0.0129	0.0022	bmi
rs17542466	A	0.0053	0.002	bmi
rs17551974	A	-0.0141	0.0022	bmi
rs17591778	A	0.0075	0.0021	bmi
rs17608150	T	0.0196	0.0031	bmi
rs17636031	T	-0.016	0.0019	bmi
rs17681451	A	-0.0197	0.0031	bmi
rs17681708	T	-0.0106	0.0018	bmi
rs17724992	A	0.0183	0.0019	bmi
rs17757975	T	0.0143	0.0024	bmi
rs17795934	T	-0.0087	0.0018	bmi
rs17806379	T	-0.0258	0.0022	bmi
rs17820822	T	0.0143	0.0018	bmi
rs1782507	T	-0.0135	0.0018	bmi
rs1789165	A	0.0138	0.0017	bmi
rs1791253	T	0.0187	0.0037	bmi
rs181732	T	-0.0019	0.0017	bmi
rs1819844	A	0.0143	0.0021	bmi
rs1852006	A	-0.0156	0.0018	bmi
rs1853639	A	-0.0111	0.0018	bmi
rs1862451	A	0.0137	0.0019	bmi
rs1865341	T	0.0128	0.002	bmi
rs1866510	T	-0.0105	0.0018	bmi
rs1884389	T	-0.0103	0.0017	bmi
rs1884897	A	-0.0194	0.0017	bmi
rs1899689	T	0.0117	0.0016	bmi
rs1899951	T	0.017	0.0024	bmi
rs1909586	T	-0.0106	0.0018	bmi
rs1927790	T	-0.0148	0.0016	bmi
rs1928295	T	0.0141	0.0016	bmi
rs1941697	A	0.0123	0.0017	bmi
rs1943477	T	-0.0198	0.0035	bmi
rs1951455	T	-0.0145	0.0019	bmi
rs1956151	A	-0.013	0.0022	bmi
rs1973993	T	-0.0208	0.0017	bmi

© 2022 Acosta JN et al. *JAMA Network Open.*

SNP	Effect Allele	Beta	SE	Trait
rs1982441	T	0.0175	0.0026	bmi
rs1983864	T	0.0158	0.0018	bmi
rs2000746	A	0.0181	0.002	bmi
rs2003616	T	0.0046	0.0019	bmi
rs200968	T	-0.0025	0.0021	bmi
rs2010281	A	-0.0161	0.0017	bmi
rs2012927	A	0.0129	0.0017	bmi
rs2030342	T	0.0143	0.0017	bmi
rs2033529	A	-0.0205	0.0018	bmi
rs2075650	A	0.0244	0.0023	bmi
rs208015	T	0.0356	0.0034	bmi
rs2080454	A	-0.0129	0.0017	bmi
rs2100814	A	0.0113	0.0017	bmi
rs2122042	T	0.0235	0.002	bmi
rs2140418	T	0.0107	0.0022	bmi
rs2143253	A	-0.0188	0.0026	bmi
rs2154297	T	-0.0115	0.0019	bmi
rs215632	A	0.0152	0.0018	bmi
rs2160077	A	0.0093	0.0016	bmi
rs2170382	T	0.0172	0.0027	bmi
rs217671	A	-0.0144	0.0019	bmi
rs2185027	A	-0.0135	0.0018	bmi
rs2190788	T	0.0141	0.0019	bmi
rs2192158	A	0.0129	0.0017	bmi
rs2194385	A	0.0091	0.0017	bmi
rs2228213	A	-0.0139	0.0017	bmi
rs2229616	T	-0.106	0.0059	bmi
rs223051	T	0.0112	0.0018	bmi
rs2230590	T	-0.0239	0.0017	bmi
rs2230929	A	-0.0048	0.0023	bmi
rs2236950	A	-0.0077	0.0022	bmi
rs2240108	T	-0.0127	0.0025	bmi
rs2275426	A	0.0104	0.0016	bmi
rs2282231	T	0.0165	0.0021	bmi
rs2283093	T	0.0127	0.0021	bmi
rs2293605	T	-0.0167	0.0027	bmi
rs2295896	A	-0.0021	0.0019	bmi
rs2304130	A	0.0176	0.003	bmi
rs2304607	A	-0.032	0.0025	bmi
rs2307022	A	0.0135	0.0017	bmi
rs2307111	T	0.0265	0.0016	bmi
rs2356865	T	-0.0114	0.0019	bmi
rs2357760	A	0.0145	0.0017	bmi
rs2365389	T	-0.0174	0.0017	bmi
rs2367112	T	0.0119	0.0016	bmi
rs2386802	A	-0.004	0.0018	bmi
rs2397061	T	-0.0094	0.0027	bmi
rs2409730	A	-0.0093	0.0017	bmi
rs2412107	T	0.0112	0.0021	bmi
rs2425241	T	-0.0197	0.0033	bmi
rs2425857	A	0.0116	0.0017	bmi
rs2429150	A	-0.0111	0.0018	bmi
rs2440885	A	0.0091	0.0017	bmi
rs2467110	T	-0.0099	0.002	bmi
rs2479958	A	0.0154	0.0018	bmi
rs248139	A	0.0133	0.0022	bmi
rs2481665	T	0.0161	0.0016	bmi
rs2491864	A	0.0137	0.0021	bmi
rs2516739	A	-0.0159	0.0021	bmi
rs2537847	A	-0.0107	0.002	bmi
rs254428	T	0.0081	0.0017	bmi
rs25832	A	0.0118	0.0019	bmi
rs2590942	T	0.0293	0.002	bmi
rs2593280	A	0.0105	0.0024	bmi
rs2605603	A	-0.0103	0.0016	bmi

© 2022 Acosta JN et al. *JAMA Network Open.*

SNP	Effect Allele	Beta	SE	Trait
rs2611742	T	-0.0152	0.0017	bmi
rs2612203	A	0.0155	0.0051	bmi
rs2619976	T	0.0104	0.0018	bmi
rs262956	T	0.0125	0.0018	bmi
rs2631681	T	-0.0113	0.0017	bmi
rs2635727	T	-0.028	0.0019	bmi
rs2653365	T	-0.0119	0.0023	bmi
rs2680648	T	0.016	0.002	bmi
rs2710323	T	-0.0141	0.0016	bmi
rs2721965	A	0.0171	0.0018	bmi
rs2724861	A	-0.0092	0.0017	bmi
rs2731222	A	0.0131	0.0019	bmi
rs2733287	A	-0.0157	0.0017	bmi
rs273504	A	-0.0153	0.0018	bmi
rs273697	A	-0.0098	0.0017	bmi
rs2768950	A	0.0116	0.0019	bmi
rs2781668	T	0.0169	0.0023	bmi
rs2814992	A	-0.0245	0.0017	bmi
rs2820311	A	-0.0235	0.0018	bmi
rs28350	A	0.0177	0.0022	bmi
rs2836961	A	-0.0102	0.0017	bmi
rs2838006	T	-0.0126	0.0018	bmi
rs284227	T	-0.0147	0.0019	bmi
rs2842385	A	-0.012	0.0022	bmi
rs2850969	T	-0.0165	0.0024	bmi
rs2863981	A	0.0077	0.0017	bmi
rs2866816	T	0.0129	0.002	bmi
rs2890652	T	-0.017	0.0023	bmi
rs2902021	T	0.0136	0.0031	bmi
rs293566	T	-0.0022	0.0018	bmi
rs2974255	A	0.0092	0.002	bmi
rs29938	T	-0.015	0.0017	bmi
rs310618	T	-0.0108	0.0018	bmi
rs3134438	A	0.0113	0.0019	bmi
rs316611	T	0.0104	0.002	bmi
rs326889	T	-0.0129	0.0018	bmi
rs329124	A	0.0131	0.0017	bmi
rs329277	T	-0.0099	0.0017	bmi
rs329651	T	0.0164	0.0021	bmi
rs331949	T	-0.011	0.0017	bmi
rs340025	T	-0.0124	0.0017	bmi
rs346722	T	-0.0027	0.0029	bmi
rs368863	T	-0.0117	0.002	bmi
rs3731544	A	0.0214	0.0031	bmi
rs3734572	T	-0.0166	0.004	bmi
rs3736485	A	0.0134	0.0016	bmi
rs3739555	T	0.011	0.0021	bmi
rs3739733	A	0.0132	0.0021	bmi
rs3746429	T	0.011	0.0023	bmi
rs3751813	T	0.0603	0.0016	bmi
rs3753549	T	-0.0203	0.0025	bmi
rs3762396	A	0.0073	0.0017	bmi
rs3768486	A	-0.0096	0.0021	bmi
rs3772934	T	0.0103	0.0019	bmi
rs3781099	T	0.021	0.0031	bmi
rs3800229	T	0.0175	0.0018	bmi
rs3800649	A	0.0122	0.0019	bmi
rs3802924	A	0.0085	0.0022	bmi
rs3803286	A	0.0181	0.0018	bmi
rs3807049	T	-0.0035	0.0019	bmi
rs3807566	T	-0.0127	0.0017	bmi
rs3808434	A	0.011	0.0016	bmi
rs3809272	A	0.0062	0.002	bmi
rs3810291	A	0.0274	0.0018	bmi
rs3814883	T	0.0232	0.0017	bmi

© 2022 Acosta JN et al. *JAMA Network Open.*

SNP	Effect Allele	Beta	SE	Trait
rs3819299	T	0.0187	0.0035	bmi
rs3821841	T	-0.0157	0.0033	bmi
rs3822683	A	0.0145	0.0021	bmi
rs3826705	T	-0.0157	0.0027	bmi
rs3829849	T	0.0098	0.0017	bmi
rs3844598	A	-0.0095	0.0017	bmi
rs3849570	A	0.0132	0.0017	bmi
rs3850422	A	-0.0114	0.0016	bmi
rs3851083	A	-0.0102	0.0016	bmi
rs3887080	A	0.0181	0.0026	bmi
rs3902840	A	0.0222	0.003	bmi
rs3902951	T	-0.0134	0.002	bmi
rs3915844	A	0.0152	0.0025	bmi
rs3922853	A	0.0123	0.0023	bmi
rs39654	A	-0.0145	0.0017	bmi
rs40067	A	-0.0266	0.0023	bmi
rs403656	A	0.0094	0.0024	bmi
rs4076358	A	0.0092	0.0017	bmi
rs4077093	T	0.0128	0.0022	bmi
rs411717	T	-0.0069	0.0017	bmi
rs4148155	A	0.0188	0.0026	bmi
rs419261	T	0.0106	0.0017	bmi
rs4273371	T	-0.0115	0.0016	bmi
rs427943	A	-0.017	0.0017	bmi
rs4284600	T	-0.0118	0.0017	bmi
rs4303732	T	0.0144	0.0017	bmi
rs4307239	A	-0.0115	0.0017	bmi
rs4339513	T	-0.0015	0.0032	bmi
rs4342060	T	-0.0051	0.0022	bmi
rs4366093	T	-0.0121	0.0018	bmi
rs4372296	A	-0.0133	0.002	bmi
rs4372836	T	0.0142	0.0018	bmi
rs4383818	T	0.0088	0.0021	bmi
rs450231	A	-0.0127	0.002	bmi
rs4515655	T	-0.0072	0.0018	bmi
rs4516268	A	-0.0217	0.0021	bmi
rs4518345	A	-0.0117	0.0019	bmi
rs4524456	A	-0.0086	0.0017	bmi
rs453520	T	-0.015	0.0017	bmi
rs4542429	T	-0.0064	0.0019	bmi
rs4624596	T	0.0136	0.0023	bmi
rs4639527	A	-0.0172	0.0019	bmi
rs4643949	T	-1.00E-04	0.0019	bmi
rs4653017	T	0.0122	0.0018	bmi
rs4670626	T	-0.0113	0.0018	bmi
rs4671328	T	0.0219	0.0017	bmi
rs4673553	T	-0.0142	0.0017	bmi
rs4676084	A	0.0098	0.0017	bmi
rs4678297	T	0.0043	0.0019	bmi
rs4718966	T	0.0127	0.0018	bmi
rs4722398	T	0.0158	0.0025	bmi
rs4722672	T	-0.0151	0.0021	bmi
rs4725984	T	-0.0128	0.0017	bmi
rs4737183	A	0.0112	0.0017	bmi
rs4740383	A	0.0126	0.0018	bmi
rs4744275	A	0.0144	0.0018	bmi
rs4759073	A	-0.0116	0.0017	bmi
rs4764949	A	0.0184	0.0018	bmi
rs4766710	A	0.0229	0.0035	bmi
rs4796243	A	-0.0124	0.0019	bmi
rs4814512	A	0.0133	0.0021	bmi
rs4820408	T	0.0151	0.0017	bmi
rs483752	T	0.0121	0.0022	bmi
rs4841659	T	0.0149	0.0017	bmi
rs4854326	A	-0.0019	0.0019	bmi

© 2022 Acosta JN et al. *JAMA Network Open.*

SNP	Effect Allele	Beta	SE	Trait
rs4858193	T	0.0129	0.0019	bmi
rs4864201	T	0.0141	0.0017	bmi
rs487060	T	0.011	0.0016	bmi
rs4886506	T	-8.00E-04	0.0019	bmi
rs4886869	A	-0.01	0.0018	bmi
rs4889782	T	-0.015	0.0018	bmi
rs4906908	T	-0.0103	0.0017	bmi
rs4916661	T	-0.0034	0.0018	bmi
rs4936175	T	-0.0122	0.0017	bmi
rs4981693	A	0.0206	0.002	bmi
rs498240	A	-0.0267	0.0033	bmi
rs4985155	A	0.012	0.0017	bmi
rs4986044	T	-0.0164	0.0016	bmi
rs524281	A	-0.0093	0.0019	bmi
rs5396	T	-0.0154	0.0018	bmi
rs543874	A	-0.0475	0.002	bmi
rs551137	T	0.0041	0.0021	bmi
rs555267	T	0.0126	0.0017	bmi
rs559267	A	-0.0115	0.0017	bmi
rs573455	A	-0.0093	0.0016	bmi
rs5750913	A	-0.0012	0.0019	bmi
rs577525	T	-0.0166	0.0017	bmi
rs580438	T	0.0114	0.0018	bmi
rs587230	A	0.0149	0.0023	bmi
rs6019482	T	-0.0178	0.0023	bmi
rs6060151	T	0.0029	0.0018	bmi
rs6061162	T	0.0081	0.003	bmi
rs6076348	A	-0.0012	0.0019	bmi
rs6088529	A	0.001	0.0018	bmi
rs6132918	T	-0.0106	0.0023	bmi
rs6138482	T	0.0147	0.002	bmi
rs6142096	A	0.0137	0.0017	bmi
rs621042	A	-0.0107	0.0017	bmi
rs6265	T	-0.0412	0.0021	bmi
rs629443	T	0.0116	0.0019	bmi
rs6442021	T	-0.009	0.0017	bmi
rs6442101	T	-0.0117	0.0017	bmi
rs6443750	T	-0.0148	0.0021	bmi
rs6448587	A	0.0167	0.0023	bmi
rs6449531	A	-0.0127	0.0018	bmi
rs6461115	A	0.0144	0.0019	bmi
rs6463489	T	0.0157	0.0026	bmi
rs6474945	T	-0.0186	0.0016	bmi
rs6477694	T	-0.0123	0.0017	bmi
rs6490055	A	-0.0072	0.002	bmi
rs6494481	T	0.012	0.0027	bmi
rs6495252	T	0.0099	0.0018	bmi
rs651548	A	0.0139	0.0018	bmi
rs6545714	A	-0.0191	0.0017	bmi
rs6548221	A	0.0151	0.002	bmi
rs6564360	A	-0.0135	0.0022	bmi
rs6569648	T	-0.0126	0.0019	bmi
rs6577584	T	-0.0131	0.0018	bmi
rs6580755	T	-0.0015	0.0018	bmi
rs6587552	A	0.0173	0.002	bmi
rs663129	A	0.0545	0.0019	bmi
rs6681627	A	0.0025	0.0025	bmi
rs6690764	A	-0.0154	0.0022	bmi
rs6700838	T	-0.017	0.0017	bmi
rs6710871	A	0.0179	0.0024	bmi
rs6711584	A	0.0128	0.0017	bmi
rs6720868	T	0.0155	0.0018	bmi
rs6738445	T	-0.0133	0.0018	bmi
rs6753170	T	-0.0031	0.0018	bmi
rs6764533	A	0.0116	0.0018	bmi

© 2022 Acosta JN et al. *JAMA Network Open.*

SNP	Effect Allele	Beta	SE	Trait
rs6786582	T	-0.0159	0.0018	bmi
rs6803870	T	0.0084	0.0024	bmi
rs6804842	A	-0.0156	0.0017	bmi
rs6818414	T	-0.0097	0.0017	bmi
rs6827083	A	-0.0097	0.0016	bmi
rs6843738	A	-0.0107	0.0017	bmi
rs6879326	T	-0.0098	0.0017	bmi
rs6900723	T	-0.0112	0.0018	bmi
rs6904676	A	0.0078	0.0021	bmi
rs6908295	A	-0.0058	0.0025	bmi
rs6919443	A	-0.0096	0.0017	bmi
rs6921533	T	0.0118	0.0019	bmi
rs6922214	A	-0.0123	0.0024	bmi
rs6922855	A	4.00E-04	0.0017	bmi
rs6938239	A	-0.0271	0.0025	bmi
rs6963840	T	0.0154	0.0024	bmi
rs696606	A	0.0095	0.0018	bmi
rs6968554	A	-0.01	0.0017	bmi
rs7006629	T	0.0109	0.0017	bmi
rs7024334	T	0.0138	0.002	bmi
rs7042372	A	0.0122	0.0018	bmi
rs7083450	T	0.0159	0.0023	bmi
rs7084454	A	0.0193	0.0019	bmi
rs7102454	T	-0.0158	0.0018	bmi
rs7117238	A	-0.0131	0.0022	bmi
rs7120873	T	0.0172	0.0027	bmi
rs7122539	A	-0.0065	0.0017	bmi
rs7123876	T	-0.0119	0.0019	bmi
rs7124442	T	-0.0286	0.0017	bmi
rs7124681	A	0.0263	0.0016	bmi
rs7133378	A	0.0125	0.0018	bmi
rs7134375	A	0.0077	0.0016	bmi
rs7138803	A	0.03	0.0017	bmi
rs7144011	T	0.0282	0.002	bmi
rs7147503	T	-0.0119	0.0018	bmi
rs7164727	T	0.0182	0.0017	bmi
rs7181498	T	0.0163	0.0018	bmi
rs7187776	A	-0.0265	0.0016	bmi
rs7195386	T	0.0133	0.0017	bmi
rs719802	T	0.0101	0.0018	bmi
rs7200919	A	0.0095	0.0017	bmi
rs7209235	A	-0.0111	0.0019	bmi
rs7211567	T	-0.0145	0.0021	bmi
rs7217226	T	-0.013	0.0017	bmi
rs7239114	A	0.0124	0.0017	bmi
rs7243357	T	0.0194	0.0021	bmi
rs7334078	T	0.0121	0.0019	bmi
rs733594	T	0.0138	0.0018	bmi
rs7377083	A	0.02	0.0018	bmi
rs740157	A	0.0116	0.0016	bmi
rs7425440	T	0.0104	0.0021	bmi
rs7444298	A	0.0179	0.002	bmi
rs7478904	T	-0.0086	0.0026	bmi
rs7531118	T	-0.0256	0.0016	bmi
rs7531656	A	0.0195	0.0017	bmi
rs7535528	A	-0.0152	0.0018	bmi
rs7550711	T	0.0649	0.005	bmi
rs7556169	A	-0.0103	0.0018	bmi
rs7557796	T	0.016	0.0018	bmi
rs7560871	A	0.0218	0.0034	bmi
rs7561278	T	0.0159	0.0021	bmi
rs7564679	A	-0.0116	0.0016	bmi
rs756717	A	-0.0148	0.0017	bmi
rs757318	A	-0.0185	0.0016	bmi
rs757608	A	-0.0092	0.0017	bmi

© 2022 Acosta JN et al. *JAMA Network Open.*

SNP	Effect Allele	Beta	SE	Trait
rs7599312	A	-0.0186	0.0019	bmi
rs7607351	T	0.0119	0.0017	bmi
rs7607369	A	0.0117	0.0016	bmi
rs7607490	A	0.0148	0.0028	bmi
rs760880	T	-0.0027	0.0017	bmi
rs761423	T	0.0113	0.0017	bmi
rs7616371	A	-0.0022	0.0033	bmi
rs762147	A	-0.0115	0.0019	bmi
rs7640424	T	-0.0136	0.0018	bmi
rs765332	T	-0.0118	0.0028	bmi
rs7674623	T	0.0135	0.0022	bmi
rs7683836	A	-0.0114	0.0017	bmi
rs7694732	A	0.0099	0.0017	bmi
rs7702514	T	-0.0016	0.0023	bmi
rs7710595	A	0.0101	0.0017	bmi
rs7711753	A	-0.0134	0.0016	bmi
rs7715256	T	-0.0166	0.0016	bmi
rs7716275	T	-0.0132	0.0021	bmi
rs7727781	T	0.0097	0.0017	bmi
rs7730004	T	0.0148	0.0018	bmi
rs7730898	A	0.0168	0.0018	bmi
rs7748777	A	0.0105	0.0016	bmi
rs775731	T	-0.0108	0.0017	bmi
rs7760082	A	-0.0122	0.0018	bmi
rs7777102	A	-0.0126	0.0024	bmi
rs7779498	T	-0.0249	0.0045	bmi
rs7784465	T	-0.0164	0.0025	bmi
rs7796608	A	0.0124	0.0025	bmi
rs7805441	T	0.0109	0.0017	bmi
rs7844647	T	0.0123	0.0018	bmi
rs7865157	T	0.0177	0.0028	bmi
rs7869771	A	0.014	0.0019	bmi
rs7874154	T	-0.0127	0.0017	bmi
rs7893571	T	0.0136	0.0018	bmi
rs7899106	A	-0.0331	0.0037	bmi
rs7903146	T	-0.0181	0.0018	bmi
rs7919	A	-0.0158	0.0017	bmi
rs7924371	T	-0.004	0.0017	bmi
rs793520	A	0.0105	0.0019	bmi
rs7941030	T	-0.0112	0.0017	bmi
rs7948120	T	-0.0132	0.0019	bmi
rs7958206	A	0.0014	0.0018	bmi
rs7965658	A	-0.0016	0.0022	bmi
rs7968230	A	0.0131	0.0018	bmi
rs7973955	A	-0.0126	0.0019	bmi
rs7975187	A	-0.0128	0.0021	bmi
rs799449	T	0.013	0.0017	bmi
rs8016771	T	-0.019	0.0031	bmi
rs8024932	T	0.0091	0.0028	bmi
rs8033510	T	0.0106	0.0018	bmi
rs8036040	A	0.0109	0.0017	bmi
rs8046061	T	0.009	0.0017	bmi
rs8047395	A	0.0642	0.0017	bmi
rs8067737	T	0.0166	0.003	bmi
rs8069296	T	-0.0104	0.002	bmi
rs8070454	T	-0.0098	0.0017	bmi
rs8071182	A	0.0133	0.0022	bmi
rs8075273	A	-0.0133	0.0018	bmi
rs8079034	T	0.0096	0.0022	bmi
rs8081039	T	0.0233	0.0038	bmi
rs8087550	A	-3.00E-04	0.0017	bmi
rs8092503	A	-0.0165	0.0019	bmi
rs8094523	A	-0.0305	0.0032	bmi
rs8097544	A	-0.02	0.0025	bmi
rs8102137	T	-0.0193	0.0018	bmi

© 2022 Acosta JN et al. *JAMA Network Open.*

SNP	Effect Allele	Beta	SE	Trait
rs8121840	A	0.0085	0.0017	bmi
rs8123881	A	-0.0196	0.0024	bmi
rs816364	A	-0.01	0.0018	bmi
rs816533	A	-0.0168	0.0047	bmi
rs820077	A	-0.0064	0.0022	bmi
rs823074	T	0.0112	0.0017	bmi
rs827092	T	0.0129	0.0017	bmi
rs845084	A	0.014	0.002	bmi
rs847747	T	-0.0113	0.0019	bmi
rs849135	A	0.0109	0.0016	bmi
rs853679	A	0.0134	0.0022	bmi
rs8567	A	-0.009	0.0016	bmi
rs874454	A	-0.0081	0.0018	bmi
rs879620	T	0.0231	0.0018	bmi
rs881301	T	-0.0097	0.0017	bmi
rs884282	T	-0.0093	0.0017	bmi
rs886444	A	-0.0102	0.0017	bmi
rs889398	T	-0.0196	0.0016	bmi
rs892261	T	-0.0103	0.0017	bmi
rs900144	T	0.0166	0.0017	bmi
rs901630	T	-0.0146	0.0017	bmi
rs902695	A	-0.0103	0.0017	bmi
rs905938	T	-0.0149	0.0019	bmi
rs907011	T	0.0102	0.0019	bmi
rs9077	A	-0.0138	0.0019	bmi
rs919433	A	0.0036	0.0018	bmi
rs925421	A	0.0116	0.002	bmi
rs926279	A	-0.0036	0.0019	bmi
rs9289499	T	-0.0031	0.0028	bmi
rs9291467	T	0.014	0.0017	bmi
rs9294260	A	0.0147	0.0016	bmi
rs929641	A	0.015	0.0016	bmi
rs9299	T	0.0121	0.0018	bmi
rs930295	A	0.0211	0.0023	bmi
rs934515	A	0.0185	0.0027	bmi
rs9349239	A	0.0122	0.0017	bmi
rs9362662	A	0.0112	0.0017	bmi
rs9367368	T	0.0121	0.0018	bmi
rs9396763	A	0.0089	0.0021	bmi
rs9419958	T	-0.0126	0.0025	bmi
rs9426003	A	-0.0116	0.0019	bmi
rs9460306	T	0.0136	0.0031	bmi
rs9463175	T	-0.0108	0.0017	bmi
rs946526	T	-0.0314	0.0043	bmi
rs9470086	A	0.0243	0.0068	bmi
rs9475173	A	0.0108	0.0019	bmi
rs9507983	T	-0.0156	0.0018	bmi
rs952159	A	-2.00E-04	0.0018	bmi
rs9527706	A	-0.0115	0.0018	bmi
rs9530843	A	0.0128	0.0018	bmi
rs9538141	A	0.0164	0.0017	bmi
rs954018	A	-0.013	0.0018	bmi
rs9540493	A	0.0139	0.0017	bmi
rs9544915	T	0.014	0.0025	bmi
rs9571687	A	-0.0129	0.0018	bmi
rs9595908	T	0.0159	0.0017	bmi
rs9603697	T	0.0141	0.0018	bmi
rs9615905	T	0.011	0.0017	bmi
rs9630985	A	-0.0177	0.0018	bmi
rs9675376	A	0.035	0.0018	bmi
rs967605	T	-0.0192	0.0023	bmi
rs9688431	T	0.0231	0.0035	bmi
rs968972	A	0.0089	0.0018	bmi
rs9714342	T	-0.0151	0.0019	bmi
rs972283	A	0.0096	0.0016	bmi

© 2022 Acosta JN et al. *JAMA Network Open.*

SNP	Effect Allele	Beta	SE	Trait
rs972540	A	-0.013	0.0018	bmi
rs977540	A	0.014	0.0019	bmi
rs9787495	A	-0.0103	0.0017	bmi
rs980329	T	-0.0133	0.002	bmi
rs9814633	A	0.0122	0.0018	bmi
rs9827072	A	-0.0114	0.0031	bmi
rs9832305	T	-0.0073	0.0019	bmi
rs9838283	A	0.0161	0.0026	bmi
rs9846123	T	-0.0011	0.0018	bmi
rs987237	A	-0.0409	0.0021	bmi
rs9881036	A	-0.0035	0.0017	bmi
rs9905991	A	0.01	0.0017	bmi
rs9922708	T	0.0692	0.0016	bmi
rs9927848	A	-0.0122	0.002	bmi
rs9931164	A	0.0245	0.0061	bmi
rs9931407	T	-8.00E-04	0.0049	bmi
rs9931967	T	0.0163	0.0017	bmi
rs993931	A	-0.0057	0.0018	bmi
rs993954	T	0.0084	0.0017	bmi
rs9951893	T	-0.0115	0.0017	bmi
rs9961813	A	0.013	0.003	bmi
rs998584	A	-0.0131	0.0017	bmi
rs998732	A	0.0171	0.0022	bmi
rs66495454	G	-0.0209469	0.0034162	diet_score
rs11412839	C	0.0230741	0.00402124	diet_score
rs6947580	A	0.0223534	0.00405973	diet_score
rs11789020	A	0.0276728	0.00335799	diet_score
rs2666769	C	-0.0220809	0.00364196	diet_score
rs3105033	A	0.0245086	0.00370567	diet_score
rs61992671	A	0.0202725	0.00343427	diet_score
rs71466817	C	-0.0242449	0.00406755	diet_score
rs1421085	T	-0.0197532	0.00335278	diet_score
rs142710267	T	0.0218579	0.00359759	diet_score
rs12969294	A	-0.019145	0.00348224	diet_score
rs564819152	A	0.028	0.005	physical_activity
rs2696625	A	-0.037	0.005	physical_activity
rs8042849	C	0.028	0.002	smoking
rs113382419	C	-0.041	0.003	smoking
rs6011779	C	0.028	0.003	smoking
rs9919670	G	-0.022	0.002	smoking
rs2890772	G	-0.02	0.002	smoking
rs35175834	G	-0.024	0.002	smoking
rs12244388	G	-0.019	0.002	smoking
rs11783093	C	0.023	0.003	smoking
rs11210229	A	0.017	0.002	smoking
rs62155874	A	-0.024	0.003	smoking
rs10226228	A	-0.016	0.002	smoking
rs6119897	G	-0.018	0.002	smoking
rs2867112	T	0.021	0.003	smoking
rs986391	G	0.016	0.002	smoking
rs3742365	T	-0.016	0.002	smoking
rs7807019	A	-0.015	0.002	smoking
rs549845	G	0.016	0.002	smoking
rs7569203	A	-0.016	0.002	smoking
rs17309874	G	-0.016	0.002	smoking
rs6778080	T	0.016	0.002	smoking
rs8042134	T	-0.014	0.002	smoking
rs17576594	G	0.016	0.002	smoking
rs7766610	C	0.018	0.003	smoking
rs1922018	C	0.014	0.002	smoking
rs7553348	G	0.014	0.002	smoking
rs7528604	G	0.014	0.002	smoking
rs329120	C	0.014	0.002	smoking
rs12623702	A	-0.014	0.002	smoking
rs13296519	G	-0.014	0.002	smoking

© 2022 Acosta JN et al. *JAMA Network Open.*

SNP	Effect Allele	Beta	SE	Trait
rs6935954	A	0.014	0.002	smoking
rs4671357	T	-0.014	0.002	smoking
rs3896224	A	0.014	0.002	smoking
rs326341	G	0.014	0.002	smoking
rs4391802	A	0.015	0.002	smoking
rs72678864	G	0.018	0.003	smoking
rs112282219	G	-0.033	0.005	smoking
rs10879871	T	-0.014	0.002	smoking
rs889398	C	0.013	0.002	smoking
rs1933270	T	0.013	0.002	smoking
rs8614	C	-0.017	0.003	smoking
rs11255908	T	-0.015	0.002	smoking
rs13153393	A	-0.02	0.003	smoking
rs7333559	G	0.015	0.002	smoking
rs76608582	C	0.031	0.005	smoking
rs421983	T	0.013	0.002	smoking
rs4543592	T	-0.012	0.002	smoking
rs11948770	T	-0.015	0.002	smoking
rs7039819	G	0.013	0.002	smoking
rs10282292	C	0.013	0.002	smoking
rs2838834	C	-0.013	0.002	smoking
rs624833	T	0.013	0.002	smoking
rs62135536	C	0.035	0.006	smoking
rs3811038	T	-0.014	0.002	smoking
rs359243	T	-0.013	0.002	smoking
rs11768481	C	0.013	0.002	smoking
rs6779302	G	-0.013	0.002	smoking
rs35169606	T	0.013	0.002	smoking
rs67596067	G	-0.013	0.002	smoking
rs2675638	G	0.012	0.002	smoking
rs75742406	G	0.014	0.002	smoking
rs71367545	G	-0.015	0.002	smoking
rs71627581	G	0.019	0.003	smoking
rs13016665	C	-0.012	0.002	smoking
rs369230	G	-0.013	0.002	smoking
rs10052591	T	0.012	0.002	smoking
rs7155595	A	-0.013	0.002	smoking
rs7077678	C	0.012	0.002	smoking
rs860326	C	0.012	0.002	smoking
rs12202536	A	-0.012	0.002	smoking
rs4814873	C	0.014	0.002	smoking
rs147412694	G	-0.017	0.003	smoking
rs9842947	C	-0.013	0.002	smoking
rs12708665	A	-0.013	0.002	smoking
rs202645	A	-0.015	0.002	smoking
rs62098013	G	-0.012	0.002	smoking
rs4957528	A	-0.015	0.002	smoking
rs1246265	T	-0.013	0.002	smoking
rs6598539	T	-0.012	0.002	smoking
rs13009008	A	0.012	0.002	smoking
rs17553262	A	-0.018	0.003	smoking
rs7297175	T	-0.012	0.002	smoking
rs245774	A	-0.013	0.002	smoking
rs6962772	A	0.016	0.003	smoking
rs35343344	C	0.013	0.002	smoking
rs2062882	G	-0.012	0.002	smoking
rs7519626	C	0.012	0.002	smoking
rs34866095	A	-0.012	0.002	smoking
rs348809	A	-0.012	0.002	smoking
rs1050847	C	0.011	0.002	smoking
rs73220544	A	-0.016	0.003	smoking
rs4571506	C	0.011	0.002	smoking
rs732083	G	0.012	0.002	smoking
rs6741228	T	0.011	0.002	smoking
rs4949465	T	-0.017	0.003	smoking

© 2022 Acosta JN et al. *JAMA Network Open.*

SNP	Effect Allele	Beta	SE	Trait
rs62175972	T	0.031	0.006	smoking
rs136233	A	-0.014	0.003	smoking
rs12831617	C	-0.013	0.002	smoking
rs11861214	G	0.014	0.002	smoking
rs10918701	G	0.012	0.002	smoking
rs74086911	G	0.021	0.004	smoking
rs4731925	C	-0.012	0.002	smoking
rs28485305	C	0.012	0.002	smoking
rs60952428	T	0.019	0.003	smoking
rs9904288	T	0.012	0.002	smoking
rs12967855	A	0.012	0.002	smoking
rs2254710	C	0.013	0.002	smoking
rs1931263	G	-0.011	0.002	smoking
rs57611503	G	0.011	0.002	smoking
rs6957896	C	-0.011	0.002	smoking
rs10087219	G	-0.007459	0.00134463	hba1c
rs10407429	G	0.00784658	0.00081871	hba1c
rs10743139	G	0.0126582	0.00183031	hba1c
rs10758593	G	-0.0051958	0.00082613	hba1c
rs10811660	G	0.0163996	0.00106545	hba1c
rs10863950	C	-0.0068776	0.00103893	hba1c
rs10873398	G	0.0066884	0.00084918	hba1c
rs10886014	C	0.00475271	0.00081108	hba1c
rs11070339	A	-0.0050494	0.00082435	hba1c
rs11166447	T	0.00510814	0.00084662	hba1c
rs11187140	G	0.00814649	0.00084556	hba1c
rs112126121	C	-0.0050688	0.00081226	hba1c
rs1121980	G	-0.0047464	0.00081991	hba1c
rs112484505	G	0.0163002	0.00293542	hba1c
rs11257655	C	-0.0112111	0.00099698	hba1c
rs112769772	T	0.0171493	0.00248164	hba1c
rs113204516	A	0.01616	0.00188057	hba1c
rs113782313	A	-0.007568	0.00084725	hba1c
rs11392438	T	0.00635176	0.00095974	hba1c
rs11454405	A	0.00494078	0.00084612	hba1c
rs115128825	C	-0.0301881	0.00286404	hba1c
rs11531378	T	-0.0087663	0.0008851	hba1c
rs11540050	G	0.0169118	0.00185936	hba1c
rs11564725	C	-0.0104733	0.00095999	hba1c
rs11597148	G	-0.0074642	0.00106413	hba1c
rs116710147	C	-0.0267123	0.00360076	hba1c
rs117031158	G	0.0231873	0.00393164	hba1c
rs117056999	C	-0.0175623	0.0022986	hba1c
rs11708067	A	0.0172589	0.0009382	hba1c
rs117108573	C	0.00966724	0.00158001	hba1c
rs117473279	G	0.024159	0.00344816	hba1c
rs11768656	C	0.0159783	0.00216726	hba1c
rs117777577	C	-0.0182714	0.00280354	hba1c
rs117817570	G	0.0249839	0.00409631	hba1c
rs11878545	G	-0.0065977	0.00114177	hba1c
rs12147688	G	-0.0075098	0.00093244	hba1c
rs12459419	C	0.00964908	0.00086236	hba1c
rs1248882	C	-0.0057941	0.00085335	hba1c
rs12611808	T	-0.0146974	0.00086618	hba1c
rs12669521	A	0.00503936	0.00085938	hba1c
rs12969025	C	0.00627186	0.00085382	hba1c
rs12985264	G	-0.0055181	0.00095551	hba1c
rs13165038	T	-0.0067058	0.00086507	hba1c
rs13192435	T	-0.0160449	0.00258648	hba1c
rs13318260	A	-0.0057662	0.00081871	hba1c
rs13327021	C	-0.0053649	0.00084865	hba1c
rs13431652	T	0.0368296	0.00088037	hba1c
rs138157637	A	0.0123173	0.00194002	hba1c
rs140395138	G	-0.014337	0.00246887	hba1c
rs141995849	G	-0.0102842	0.00171225	hba1c

© 2022 Acosta JN et al. *JAMA Network Open.*

SNP	Effect Allele	Beta	SE	Trait
rs142391093	A	-0.0055958	0.00095399	hba1c
rs143667358	TATACATACATAC	-0.0089751	0.00103178	hba1c
rs143777421	C	-0.0109994	0.00185816	hba1c
rs144192755	C	0.023549	0.00385679	hba1c
rs145016347	T	-0.0132049	0.00238984	hba1c
rs146302237	A	0.0114036	0.00120812	hba1c
rs146933081	C	0.0237472	0.00404858	hba1c
rs147001976	G	0.0176273	0.00227116	hba1c
rs148644119	A	0.00891125	0.00151999	hba1c
rs149966	G	0.00923577	0.00123358	hba1c
rs1508615	T	0.00464106	0.00081093	hba1c
rs151059905	A	-0.0154707	0.00255359	hba1c
rs1579238	A	0.00648505	0.00094589	hba1c
rs1604038	C	0.0146913	0.00089546	hba1c
rs1611236	A	-0.0063102	0.00087261	hba1c
rs17036143	G	-0.0088828	0.00120033	hba1c
rs17122779	A	-0.0052645	0.00096248	hba1c
rs17168486	C	-0.0090754	0.00107321	hba1c
rs17462188	C	-0.0059203	0.00088182	hba1c
rs17508261	T	-0.0101772	0.00123903	hba1c
rs17850433	T	-0.0390165	0.00369966	hba1c
rs184499898	A	-0.0285497	0.00401145	hba1c
rs1866476	T	0.00521788	0.00091545	hba1c
rs1977383	T	-0.0051101	0.0008199	hba1c
rs1983128	G	-0.0049944	0.00088584	hba1c
rs200118455	TG	-0.0261166	0.00091231	hba1c
rs201539436	TA	-0.0068889	0.00106195	hba1c
rs2071053	A	-0.0061389	0.00091672	hba1c
rs2168101	C	0.00594654	0.00090032	hba1c
rs2220001	T	-0.0052358	0.00085763	hba1c
rs2237895	A	-0.0071953	0.0008193	hba1c
rs2244566	A	0.00732556	0.0011256	hba1c
rs2248933	G	0.00602855	0.00095617	hba1c
rs2286480	C	-0.005634	0.00086566	hba1c
rs231360	C	-0.0047772	0.00083185	hba1c
rs2389615	T	-0.0116569	0.00121938	hba1c
rs2390730	C	-0.0088838	0.00117187	hba1c
rs258222	C	0.00594783	0.00081375	hba1c
rs2583934	G	-0.0074651	0.00117584	hba1c
rs2812541	A	-0.0061718	0.00081364	hba1c
rs28378473	T	-0.01119	0.00091977	hba1c
rs28483077	A	-0.0071585	0.0010883	hba1c
rs28483956	G	-0.0107599	0.00195258	hba1c
rs28565268	T	-0.0118843	0.00187044	hba1c
rs28659953	T	-0.0060965	0.00083788	hba1c
rs28663084	G	0.00724059	0.00086304	hba1c
rs2966083	G	0.00483846	0.0008166	hba1c
rs2973342	A	-0.0053966	0.00093701	hba1c
rs2987456	T	-0.0103649	0.00175132	hba1c
rs3217860	A	-0.0074348	0.00094244	hba1c
rs34592828	G	0.0109527	0.00194033	hba1c
rs34865291	A	-0.0096369	0.00175796	hba1c
rs35024453	C	-0.0065645	0.00117331	hba1c
rs35537689	G	-0.0050374	0.00088029	hba1c
rs35660593	C	-0.0069823	0.00120098	hba1c
rs35781149	C	0.0138094	0.00222402	hba1c
rs35849071	CA	-0.0097054	0.00166425	hba1c
rs368865	A	-0.01205	0.00090619	hba1c
rs370079421	A	-0.0063159	0.00092132	hba1c
rs372855300	A	0.00757471	0.0011406	hba1c
rs373130596	CTATT	0.011581	0.00210347	hba1c
rs374017936	T	-0.0060671	0.00092228	hba1c
rs3757970	A	-0.0059419	0.00083309	hba1c
rs3784634	C	0.00467549	0.0008162	hba1c
rs3789586	C	0.00900028	0.00112709	hba1c

© 2022 Acosta JN et al. *JAMA Network Open.*

SNP	Effect Allele	Beta	SE	Trait
rs3948593	T	0.00604334	0.00110639	hba1c
rs4418728	G	0.00715905	0.00081286	hba1c
rs4473913	C	0.00616237	0.00080896	hba1c
rs4779054	A	-0.0054828	0.00085057	hba1c
rs4820059	G	0.00606468	0.00082452	hba1c
rs492699	T	-0.0062829	0.00100893	hba1c
rs543159	C	0.00563642	0.00081306	hba1c
rs55689310	G	-0.01902	0.00328001	hba1c
rs55859594	C	-0.0107705	0.00165004	hba1c
rs56087308	A	-0.0158572	0.00224877	hba1c
rs56233660	G	-0.014798	0.0014659	hba1c
rs576674	G	0.00953054	0.0010867	hba1c
rs61138219	C	0.00905459	0.00112808	hba1c
rs61735313	G	0.0189223	0.00278076	hba1c
rs61944004	G	-0.0133892	0.00098164	hba1c
rs61990729	C	0.00847218	0.00096261	hba1c
rs62019188	G	0.00484684	0.00082192	hba1c
rs636089	T	-0.0061393	0.00083269	hba1c
rs6460094	A	0.0049888	0.00082627	hba1c
rs6772129	A	0.00550575	0.00089165	hba1c
rs6777684	A	-0.0069855	0.000833	hba1c
rs68112593	C	-0.0056883	0.000945	hba1c
rs6953344	A	-0.0052957	0.00094166	hba1c
rs6973494	C	-0.0062009	0.00082068	hba1c
rs6980437	T	-0.0079936	0.00081339	hba1c
rs71274822	C	0.00848394	0.00100962	hba1c
rs71430652	T	-0.0191844	0.00119664	hba1c
rs7151822	T	0.00695554	0.00094087	hba1c
rs7195055	C	0.0196693	0.0033795	hba1c
rs7222851	T	0.00502366	0.00081146	hba1c
rs72729575	T	-0.0170506	0.00299882	hba1c
rs727405	G	0.00472987	0.00081156	hba1c
rs72790097	T	-0.013231	0.00236343	hba1c
rs72807721	C	-0.0206079	0.00280684	hba1c
rs72814215	G	0.0251394	0.0038646	hba1c
rs73032206	A	-0.0258412	0.00230555	hba1c
rs73038369	G	0.00542426	0.00082591	hba1c
rs73082037	C	0.00602129	0.00088941	hba1c
rs73129515	G	-0.0069665	0.00115598	hba1c
rs7317848	T	-0.0113835	0.002023	hba1c
rs73368485	G	-0.0084995	0.00149397	hba1c
rs7399581	T	-0.0081731	0.00126883	hba1c
rs74598960	T	0.0139938	0.00174456	hba1c
rs75367758	G	0.0152418	0.00188131	hba1c
rs7546017	C	-0.0065501	0.00093159	hba1c
rs757110	C	0.0060418	0.00084461	hba1c
rs7603054	G	0.00591881	0.00089813	hba1c
rs76323047	A	-0.0200279	0.00125947	hba1c
rs7681002	A	0.00497579	0.00081228	hba1c
rs76957877	C	0.00622889	0.00093118	hba1c
rs77020674	C	0.0147205	0.0025492	hba1c
rs7727635	G	0.00657221	0.00112995	hba1c
rs78152251	T	0.00844015	0.00093885	hba1c
rs7903146	C	-0.0180721	0.0008942	hba1c
rs79509806	C	-0.0203342	0.00262044	hba1c
rs860262	C	0.00792196	0.00080888	hba1c
rs879619	G	-0.0070406	0.00118807	hba1c
rs882834	C	-0.0084612	0.0011643	hba1c
rs920356	T	0.0060836	0.00091045	hba1c
rs925095	C	0.00569843	0.00083625	hba1c
rs9302377	C	-0.0060367	0.00085733	hba1c
rs9462407	T	-0.0076003	0.00128293	hba1c
rs9903102	A	0.00892206	0.00115977	hba1c
rs10062361	T	0.0245367	0.00333564	ldl
rs10065787	T	-0.0189185	0.00316164	ldl

© 2022 Acosta JN et al. *JAMA Network Open.*

SNP	Effect Allele	Beta	SE	Trait
rs10119	A	0.0667509	0.00433749	ldl
rs1014283	A	-0.0186	0.00310014	ldl
rs10185855	A	0.0138	0.00240009	ldl
rs10263252	A	-0.0229504	0.003339	ldl
rs1044418	T	0.018036	0.00280011	ldl
rs10455872	A	-0.0876489	0.00566161	ldl
rs1048013	T	0.0122763	0.00200274	ldl
rs10489488	A	-0.093381	0.0111432	ldl
rs10490626	A	-0.0446992	0.00390389	ldl
rs10513551	T	-0.0154	0.00240013	ldl
rs10757272	T	-0.0195417	0.00310026	ldl
rs10773003	A	0.0237123	0.00400018	ldl
rs10874746	T	-0.0154	0.0021001	ldl
rs10893500	T	-0.042681	0.00340089	ldl
rs11080150	A	0.0147611	0.00220851	ldl
rs11125936	T	0.0245	0.00330016	ldl
rs11206510	T	0.0684207	0.00305217	ldl
rs1122608	T	-0.0478847	0.00286419	ldl
rs1129187	T	-0.0122723	0.00200056	ldl
rs1135062	A	-0.0168939	0.00233334	ldl
rs11571836	A	0.0212652	0.00340279	ldl
rs11583680	T	0.027229	0.00386538	ldl
rs115881343	T	0.194533	0.00946356	ldl
rs11603023	T	0.0125951	0.00200009	ldl
rs1165222	A	-0.135172	0.00803552	ldl
rs11669338	T	0.0501229	0.00622267	ldl
rs11669576	A	0.0636435	0.00657363	ldl
rs1169288	A	-0.034589	0.00220066	ldl
rs11782386	T	-0.0245992	0.00343943	ldl
rs118004742	T	0.0291145	0.00486936	ldl
rs12175867	T	0.0254615	0.0033225	ldl
rs12601110	A	0.0271828	0.00464639	ldl
rs1260327	A	0.0148061	0.00221016	ldl
rs12610605	A	0.126176	0.00462269	ldl
rs12670798	T	-0.0294581	0.00240168	ldl
rs12710745	A	0.0200235	0.00258844	ldl
rs12742537	A	0.015254	0.00200105	ldl
rs12748152	T	0.0260175	0.00380063	ldl
rs129128	T	0.0207817	0.0035157	ldl
rs12939848	T	-0.013991	0.00240104	ldl
rs13192471	T	-0.0336022	0.00280957	ldl
rs13268	A	0.0420195	0.00670076	ldl
rs13315871	A	-0.0322613	0.00360027	ldl
rs138777	A	0.0125422	0.00210022	ldl
rs1475701	T	-0.0676391	0.00635411	ldl
rs1487971	T	-0.0156778	0.00241364	ldl
rs1521516	T	-0.0161	0.00250013	ldl
rs157580	A	0.0754043	0.003332	ldl
rs17111483	T	0.0403408	0.00490323	ldl
rs1730859	A	-0.019016	0.00260026	ldl
rs174449	A	0.0267568	0.00250165	ldl
rs17651629	T	-0.031728	0.00371295	ldl
rs17789218	T	0.0219744	0.00270035	ldl
rs1800562	A	-0.0492004	0.00440972	ldl
rs1808458	T	0.0309871	0.00490454	ldl
rs1891110	A	0.0212	0.0020002	ldl
rs1982074	A	0.022938	0.00263581	ldl
rs2143544	T	-0.0164931	0.00282773	ldl
rs217181	T	-0.0493884	0.0033711	ldl
rs2183573	A	-0.0143811	0.0023001	ldl
rs2235215	T	0.0245663	0.00280049	ldl
rs2239619	A	0.015877	0.00210021	ldl
rs2249741	A	-0.0202937	0.00271563	ldl
rs2251219	T	-0.013669	0.00210009	ldl
rs2282889	A	-0.0163803	0.00280178	ldl

© 2022 Acosta JN et al. *JAMA Network Open.*

SNP	Effect Allele	Beta	SE	Trait
rs2287623	A	-0.0181	0.00200014	ldl
rs2391211	T	0.0230628	0.00354426	ldl
rs2479409	A	-0.0224544	0.00228986	ldl
rs2521567	A	-0.0148666	0.00240093	ldl
rs2522056	A	-0.0181503	0.00244776	ldl
rs2569538	A	-0.0606404	0.00655015	ldl
rs2737245	T	-0.0178573	0.00300035	ldl
rs2745865	T	0.0386579	0.00363607	ldl
rs2833487	A	-0.0349539	0.00560029	ldl
rs28555129	A	0.0134	0.00220008	ldl
rs2886232	T	0.0409358	0.00490021	ldl
rs28929474	T	0.0713	0.00800059	ldl
rs2954038	A	-0.0368035	0.00313644	ldl
rs2992753	A	-0.0123677	0.00210028	ldl
rs314253	T	0.0202827	0.00210031	ldl
rs34130495	A	0.0477932	0.00653027	ldl
rs35106910	A	0.0317128	0.00468627	ldl
rs35172831	T	0.0191442	0.00280035	ldl
rs35350976	A	-0.0167819	0.00290031	ldl
rs3742318	T	0.020109	0.00250203	ldl
rs3745677	A	0.0774449	0.00842967	ldl
rs3780181	A	0.0328	0.00390025	ldl
rs3798220	T	-0.136335	0.00803128	ldl
rs3798236	T	0.0184	0.00250017	ldl
rs3800406	A	0.0277676	0.00390167	ldl
rs3812594	A	-0.0138795	0.00230008	ldl
rs3812945	T	-0.015	0.00240012	ldl
rs3816492	T	-0.0167127	0.00240062	ldl
rs3816873	T	0.0135245	0.00230068	ldl
rs3846662	A	-0.0565264	0.00231751	ldl
rs3852856	A	0.0611537	0.00474829	ldl
rs3852861	T	-0.0563322	0.00397141	ldl
rs405509	T	0.172689	0.00449913	ldl
rs4077440	T	0.116651	0.00495193	ldl
rs4129767	A	0.0156634	0.00200155	ldl
rs4148218	A	-0.0597786	0.00338387	ldl
rs4300767	A	-0.0947333	0.00564756	ldl
rs4302748	A	0.0152162	0.00250017	ldl
rs4485425	A	-0.0188995	0.00230137	ldl
rs4530754	A	0.0161016	0.00210017	ldl
rs4635554	T	-0.0220509	0.00252958	ldl
rs4704825	A	-0.0279	0.00270043	ldl
rs4752805	A	-0.0148	0.00240009	ldl
rs4773173	A	0.0164	0.00250013	ldl
rs4804579	T	0.0243989	0.00386774	ldl
rs4921914	T	-0.0180873	0.00240023	ldl
rs492602	A	-0.0278638	0.00210231	ldl
rs4968318	A	0.0220449	0.00202897	ldl
rs4968839	T	0.0387009	0.00268893	ldl
rs515135	T	-0.0882491	0.00290582	ldl
rs562556	A	0.129849	0.00787807	ldl
rs58542926	T	-0.0978271	0.00380395	ldl
rs5927	A	-0.0298673	0.00384446	ldl
rs6016373	A	0.0226957	0.00200294	ldl
rs6058302	T	-0.027054	0.00340178	ldl
rs6072328	T	-0.0263578	0.00270154	ldl
rs61754230	T	0.0515	0.00770031	ldl
rs62122515	A	0.025823	0.00345816	ldl
rs62440901	T	0.0357738	0.0038363	ldl
rs641738	T	0.0140545	0.00230231	ldl
rs6502640	A	0.0194769	0.00330045	ldl
rs6511721	A	-0.0371375	0.00317007	ldl
rs6511727	T	0.0201858	0.00253825	ldl
rs6547409	T	-0.0953301	0.00574796	ldl
rs6706968	A	-0.0228036	0.00280041	ldl

© 2022 Acosta JN et al. *JAMA Network Open.*

SNP	Effect Allele	Beta	SE	Trait
rs6718187	A	-0.069694	0.00504365	ldl
rs67710536	A	-0.0267367	0.0034002	ldl
rs679899	A	-0.0294724	0.00232304	ldl
rs6859	A	0.0782996	0.00320536	ldl
rs693668	A	0.0285942	0.00364661	ldl
rs704	A	0.0200843	0.00201595	ldl
rs7080366	T	0.0194	0.00250021	ldl
rs72796748	T	0.0587622	0.00654076	ldl
rs72852601	T	0.0480584	0.0080654	ldl
rs73015030	A	-0.0787604	0.00859121	ldl
rs732841	A	0.0412535	0.00572866	ldl
rs7551981	T	0.0296484	0.0027626	ldl
rs7616006	A	0.0222	0.00240026	ldl
rs7640978	T	-0.0321	0.00350027	ldl
rs769450	A	0.0604545	0.00473904	ldl
rs77542162	A	-0.17669	0.00907421	ldl
rs8103315	A	0.0276654	0.00412636	ldl
rs8104483	T	-0.087365	0.00381274	ldl
rs867772	A	-0.0258	0.00290036	ldl
rs870992	A	-0.0268	0.00420021	ldl
rs871841	T	-0.014636	0.00200027	ldl
rs887829	T	-0.0205	0.00210018	ldl
rs892115	T	-0.0232211	0.00366404	ldl
rs926663	A	0.014434	0.0025047	ldl
rs9298506	A	-0.0217306	0.00270022	ldl
rs9302635	T	0.0550402	0.00408136	ldl
rs9376090	T	0.0275159	0.00230029	ldl
rs9390698	A	0.0128635	0.00200017	ldl
rs941408	T	0.0163056	0.00260138	ldl
rs9646133	T	-0.0194514	0.00210017	ldl
rs11774381	T	-0.0286323	0.00280662	ldl
rs12208357	T	0.0615833	0.00404665	ldl
rs12602912	T	0.0156499	0.00240161	ldl
rs12740374	T	-0.159789	0.00240958	ldl
rs13107325	T	-0.0292482	0.00410128	ldl
rs13379043	T	0.0144281	0.0022001	ldl
rs1473886	T	-0.0146245	0.00230062	ldl
rs157582	T	-0.0323509	0.00496632	ldl
rs1652507	T	0.0269912	0.00297497	ldl
rs1997243	A	-0.0164304	0.00283311	ldl
rs2068888	A	-0.0171231	0.00200013	ldl
rs2081687	T	0.026817	0.00210033	ldl
rs2792751	T	0.0262	0.00220027	ldl
rs3184504	T	-0.0257673	0.00210043	ldl
rs3748034	T	0.0179	0.0030001	ldl
rs3905000	A	-0.0179	0.0029001	ldl
rs439401	T	0.0291891	0.00302243	ldl
rs4722551	T	-0.0386137	0.00283571	ldl
rs558971	A	-0.0363	0.00240071	ldl
rs59325138	T	0.0981579	0.00421935	ldl
rs6062343	A	-0.0146	0.00200009	ldl
rs635634	T	0.0749714	0.00260206	ldl
rs6435161	T	0.0235939	0.00250359	ldl
rs7241918	T	0.0191024	0.00280022	ldl
rs72836561	T	-0.0373312	0.00590066	ldl
rs77960347	A	-0.0796773	0.00930093	ldl
rs7941030	T	-0.0137759	0.00200022	ldl
rs814295	A	0.0305243	0.00336567	ldl
rs1000423	T	0.4138	0.0346	sbp
rs10008637	T	0.2157	0.0302	sbp
rs10048404	T	-0.2607	0.0317	sbp
rs1006545	T	0.6846	0.048	sbp
rs10069690	T	0.3098	0.0369	sbp
rs10091532	A	-0.2067	0.0305	sbp
rs1010064	A	0.3571	0.0387	sbp

© 2022 Acosta JN et al. *JAMA Network Open.*

SNP	Effect Allele	Beta	SE	Trait
rs10188003	T	0.1883	0.0307	sbp
rs10207726	T	-0.2142	0.033	sbp
rs10224210	T	-0.3831	0.034	sbp
rs10282122	T	-0.302	0.0327	sbp
rs10420519	T	-0.4921	0.0887	sbp
rs1043069	T	0.234	0.0311	sbp
rs1044822	T	-0.248	0.0424	sbp
rs10460108	A	0.2141	0.0301	sbp
rs10501410	A	0.4122	0.0607	sbp
rs1052501	T	0.2262	0.0412	sbp
rs10749572	T	-0.203	0.0302	sbp
rs10776752	T	0.8211	0.0576	sbp
rs10777213	A	-0.1786	0.0299	sbp
rs10779795	A	0.2191	0.032	sbp
rs10782230	A	0.2106	0.0302	sbp
rs10804330	T	0.2351	0.0306	sbp
rs10941043	T	-0.2585	0.0332	sbp
rs10980408	T	-0.7606	0.0827	sbp
rs11097909	T	-0.3628	0.043	sbp
rs11120093	T	-0.1792	0.0307	sbp
rs11145807	A	0.2135	0.0322	sbp
rs11159091	A	0.1978	0.0303	sbp
rs111866816	T	0.3569	0.0597	sbp
rs11191580	T	1.0995	0.055	sbp
rs11210029	A	-0.203	0.0313	sbp
rs11241313	T	-0.2071	0.0326	sbp
rs11252324	T	-0.4164	0.0573	sbp
rs113086489	T	0.3249	0.0307	sbp
rs1133400	A	-0.2975	0.0376	sbp
rs113695818	T	-0.1835	0.033	sbp
rs1154214	T	-0.2031	0.0306	sbp
rs11592107	A	0.3024	0.0326	sbp
rs11604310	T	-0.2778	0.0411	sbp
rs11636952	T	0.5313	0.0328	sbp
rs11641374	A	-0.1943	0.0309	sbp
rs11655604	T	-0.2033	0.0333	sbp
rs11672660	T	0.2212	0.0381	sbp
rs11694601	A	-0.1909	0.0309	sbp
rs117206641	T	0.3154	0.0499	sbp
rs117464403	A	0.864	0.1199	sbp
rs11874246	T	0.2856	0.0328	sbp
rs11925504	A	-0.2901	0.0305	sbp
rs11960210	T	0.4727	0.0313	sbp
rs11977526	A	-0.3213	0.0312	sbp
rs1199330	A	-0.2654	0.047	sbp
rs12042924	T	-0.1807	0.0303	sbp
rs12063372	A	0.1989	0.0318	sbp
rs1209384	A	0.2558	0.0313	sbp
rs12136922	A	0.2027	0.0304	sbp
rs12153395	A	-0.3303	0.0486	sbp
rs12255372	T	0.2358	0.0335	sbp
rs12264186	T	0.2135	0.0387	sbp
rs12426261	A	0.3775	0.0309	sbp
rs12446456	T	-0.3003	0.0302	sbp
rs12464602	A	-0.2437	0.0315	sbp
rs12509595	T	-0.8367	0.0334	sbp
rs12511987	T	-0.2329	0.0399	sbp
rs12596630	T	0.4278	0.0547	sbp
rs12627651	A	0.3498	0.0341	sbp
rs12637573	A	-0.1731	0.0302	sbp
rs12643599	A	0.3134	0.0313	sbp
rs12656497	T	-0.6382	0.0307	sbp
rs12661036	T	-0.2104	0.0374	sbp
rs12668436	T	-0.2151	0.035	sbp
rs12693982	T	0.2575	0.0309	sbp

© 2022 Acosta JN et al. *JAMA Network Open.*

SNP	Effect Allele	Beta	SE	Trait
rs12694277	T	-0.2018	0.0335	sbp
rs12731646	T	-0.189	0.0307	sbp
rs1275988	T	-0.541	0.0308	sbp
rs12883810	T	-0.2382	0.0428	sbp
rs12885878	A	-0.2291	0.0367	sbp
rs12906962	T	-0.2653	0.0325	sbp
rs1290784	T	0.4124	0.0303	sbp
rs1290933	A	-0.2847	0.0327	sbp
rs12926550	A	-0.2548	0.0324	sbp
rs1293969	T	-0.1988	0.0347	sbp
rs12985940	T	0.4642	0.0434	sbp
rs13016772	T	0.2522	0.0355	sbp
rs13107261	A	-0.1778	0.0314	sbp
rs13107325	T	-0.9086	0.0592	sbp
rs13179413	T	0.2238	0.0347	sbp
rs13204703	T	0.1967	0.035	sbp
rs13253358	T	0.2127	0.033	sbp
rs13289468	A	0.2488	0.0306	sbp
rs1332813	T	0.2203	0.0314	sbp
rs13358657	A	-0.388	0.0445	sbp
rs1340030	T	0.1936	0.0312	sbp
rs13412750	A	-0.2889	0.0341	sbp
rs13420463	A	0.3143	0.036	sbp
rs1375564	T	0.2579	0.0315	sbp
rs1382472	A	-0.1917	0.0307	sbp
rs139354822	T	0.6115	0.0975	sbp
rs1408945	T	-0.3196	0.0304	sbp
rs1410222	T	0.2173	0.0388	sbp
rs1433121	T	-0.228	0.0326	sbp
rs1436138	A	0.3119	0.0315	sbp
rs1437649	A	-0.2189	0.0357	sbp
rs146550789	T	-0.4824	0.0778	sbp
rs148140538	T	-0.3252	0.0562	sbp
rs148401029	A	-0.4623	0.0848	sbp
rs1493132	T	-0.1766	0.0318	sbp
rs149339216	T	-0.6912	0.0779	sbp
rs1551355	T	0.2098	0.0356	sbp
rs1565440	A	0.1746	0.0311	sbp
rs1575290	T	0.1973	0.0301	sbp
rs1623474	T	0.3827	0.0321	sbp
rs1624822	T	-0.3362	0.0312	sbp
rs1630736	T	-0.1706	0.0309	sbp
rs167479	T	-0.5642	0.0327	sbp
rs17010957	T	-0.534	0.043	sbp
rs17035181	T	0.3074	0.0429	sbp
rs17245822	A	-0.1899	0.0312	sbp
rs17249754	A	-0.8446	0.0403	sbp
rs17257081	A	0.2274	0.0392	sbp
rs17562391	T	0.1967	0.0306	sbp
rs17608766	T	-0.6903	0.0433	sbp
rs17684859	T	-0.2241	0.034	sbp
rs17760259	T	-0.2654	0.0304	sbp
rs17762	A	0.4117	0.0571	sbp
rs17807723	A	-0.2721	0.0443	sbp
rs17812022	T	-0.3613	0.0525	sbp
rs1814951	A	-0.3231	0.0466	sbp
rs1871190	T	0.1954	0.0324	sbp
rs1882212	A	0.2753	0.0363	sbp
rs1882961	T	0.2443	0.0326	sbp
rs1889785	A	0.1782	0.0304	sbp
rs1896326	A	-0.2797	0.0371	sbp
rs1906672	A	0.2966	0.0358	sbp
rs1957563	T	0.3629	0.0342	sbp
rs1984195	A	0.2409	0.0303	sbp
rs1994158	A	0.2513	0.0391	sbp

© 2022 Acosta JN et al. *JAMA Network Open.*

SNP	Effect Allele	Beta	SE	Trait
rs2014408	T	0.5169	0.0373	sbp
rs2111557	T	0.1764	0.0302	sbp
rs2113077	A	0.2097	0.0305	sbp
rs2126474	T	-0.2601	0.0306	sbp
rs2161967	T	0.2836	0.0307	sbp
rs2177843	T	0.4394	0.0432	sbp
rs2236295	T	-0.3028	0.0309	sbp
rs2238787	A	0.2552	0.0332	sbp
rs2249105	A	0.2927	0.0313	sbp
rs2289124	A	-0.308	0.0415	sbp
rs2291434	T	-0.2622	0.0303	sbp
rs2306363	T	-0.4358	0.0376	sbp
rs234623	A	-0.1804	0.0302	sbp
rs2353940	T	-0.2075	0.0358	sbp
rs2354862	A	0.2507	0.0317	sbp
rs2384063	T	0.3266	0.0357	sbp
rs2392929	T	-0.7507	0.0379	sbp
rs2423514	A	0.3011	0.0302	sbp
rs246973	T	0.2479	0.0335	sbp
rs2470004	T	-0.3454	0.0392	sbp
rs2493296	T	0.4183	0.0442	sbp
rs2498323	A	0.3171	0.0517	sbp
rs2580350	A	0.1769	0.0307	sbp
rs2589218	T	-0.2258	0.0339	sbp
rs2596498	T	-0.233	0.0337	sbp
rs2598	A	0.168	0.0303	sbp
rs2610990	A	-0.2903	0.0343	sbp
rs2627313	T	0.3208	0.0303	sbp
rs262986	A	-0.2371	0.0305	sbp
rs263532	T	0.1798	0.0307	sbp
rs2643826	T	0.4473	0.0306	sbp
rs2652812	T	-0.2516	0.0353	sbp
rs2689690	T	-0.2702	0.0316	sbp
rs2724377	A	0.1938	0.0301	sbp
rs2745599	A	0.2164	0.0317	sbp
rs2776037	T	-0.1851	0.0309	sbp
rs2833834	A	0.2177	0.0338	sbp
rs28365916	T	-0.1713	0.0306	sbp
rs28374392	T	0.1924	0.0338	sbp
rs28429256	A	0.215	0.0325	sbp
rs28572357	A	-0.2733	0.0308	sbp
rs28578714	T	0.2066	0.0327	sbp
rs28688791	T	-0.3222	0.038	sbp
rs28866311	T	-0.2762	0.0302	sbp
rs2904315	A	-0.2081	0.0325	sbp
rs2913920	T	0.2418	0.0359	sbp
rs3098186	T	-0.2422	0.0303	sbp
rs3132442	T	0.3931	0.0304	sbp
rs33836	T	0.1766	0.0304	sbp
rs34025993	A	0.223	0.0308	sbp
rs34072724	A	-0.2422	0.0303	sbp
rs34079867	T	0.1992	0.0354	sbp
rs34130368	T	-0.3016	0.0497	sbp
rs34487963	A	-0.8819	0.1244	sbp
rs34496659	A	0.4545	0.0616	sbp
rs34535756	T	0.478	0.0786	sbp
rs34727427	T	-0.2353	0.0324	sbp
rs34941092	A	-0.3225	0.0425	sbp
rs35098810	A	0.1967	0.0356	sbp
rs35413927	A	-0.3002	0.0328	sbp
rs35444	A	0.4368	0.031	sbp
rs35680304	T	0.2694	0.031	sbp
rs35783704	A	-0.4619	0.0507	sbp
rs360153	T	-0.3445	0.0306	sbp
rs365990	A	0.225	0.0312	sbp

© 2022 Acosta JN et al. *JAMA Network Open.*

SNP	Effect Allele	Beta	SE	Trait
rs3735533	T	-0.91	0.0577	sbp
rs3764400	T	0.3748	0.0445	sbp
rs3772219	A	0.2733	0.0324	sbp
rs3807925	A	-0.1859	0.0319	sbp
rs3816865	A	0.3092	0.0565	sbp
rs3819532	T	-0.1875	0.0306	sbp
rs3860770	A	-0.2663	0.0333	sbp
rs3918226	T	0.664	0.0575	sbp
rs3950627	A	0.1851	0.0308	sbp
rs3980686	T	-0.4998	0.0487	sbp
rs404100	T	0.1935	0.0303	sbp
rs4143175	T	0.2187	0.0352	sbp
rs42032	A	-0.3231	0.0345	sbp
rs4245599	A	-0.1794	0.0305	sbp
rs4274337	A	-0.2968	0.0406	sbp
rs4286632	A	0.211	0.0343	sbp
rs4319878	T	0.1694	0.0308	sbp
rs4408839	A	-0.2301	0.0345	sbp
rs4440615	A	-0.2201	0.0312	sbp
rs4511593	T	-0.2881	0.0318	sbp
rs4553000	T	-0.2035	0.03	sbp
rs4577304	T	-0.1767	0.0302	sbp
rs4606697	A	-0.3196	0.0523	sbp
rs4651224	T	0.1986	0.0306	sbp
rs4667454	A	0.2636	0.0322	sbp
rs4775769	T	-0.4162	0.0517	sbp
rs4784541	T	-0.2015	0.0307	sbp
rs483071	T	0.2709	0.0313	sbp
rs4838021	T	-0.3009	0.0453	sbp
rs4873492	T	0.3431	0.0403	sbp
rs488834	T	-0.3799	0.0365	sbp
rs4888408	A	0.3653	0.0307	sbp
rs4908348	T	0.2366	0.033	sbp
rs4925159	A	0.2174	0.0305	sbp
rs4932373	A	-0.635	0.0328	sbp
rs4948643	T	0.2258	0.0338	sbp
rs4952609	A	0.2124	0.0347	sbp
rs4955575	A	0.2158	0.0348	sbp
rs4957026	A	0.1982	0.0323	sbp
rs4961293	T	0.2268	0.0303	sbp
rs509833	A	0.329	0.044	sbp
rs55924432	T	0.2651	0.0317	sbp
rs55944332	A	-0.2613	0.0355	sbp
rs56288724	A	-0.2178	0.031	sbp
rs56407827	T	0.3603	0.034	sbp
rs569550	T	-0.5765	0.0318	sbp
rs571689	T	0.228	0.0304	sbp
rs57342147	A	0.2787	0.0508	sbp
rs573455	A	0.1994	0.0303	sbp
rs5742643	T	-0.2233	0.0349	sbp
rs57786342	A	0.2317	0.0374	sbp
rs57946343	T	0.716	0.0426	sbp
rs59980837	T	1.0997	0.1163	sbp
rs60191654	A	-0.2382	0.0385	sbp
rs6029756	A	-0.2712	0.033	sbp
rs6031431	A	-0.2617	0.0304	sbp
rs604723	T	-0.655	0.0339	sbp
rs6054139	A	0.2094	0.0306	sbp
rs6058088	T	0.2832	0.0417	sbp
rs6062324	A	-0.3294	0.0363	sbp
rs6078093	A	-0.1849	0.0304	sbp
rs6090907	A	-0.3854	0.0425	sbp
rs60991988	T	0.3789	0.0498	sbp
rs6108787	T	-0.4274	0.03	sbp
rs61772592	A	-0.3181	0.0455	sbp

© 2022 Acosta JN et al. *JAMA Network Open.*

SNP	Effect Allele	Beta	SE	Trait
rs61917655	T	0.3427	0.0514	sbp
rs62047964	T	0.5115	0.0686	sbp
rs62076622	A	0.2363	0.0377	sbp
rs62170470	T	0.1972	0.0321	sbp
rs62187653	T	0.3286	0.0511	sbp
rs62309747	A	-0.2244	0.0304	sbp
rs6271	T	-0.5547	0.0611	sbp
rs641620	T	-0.3193	0.044	sbp
rs6438857	T	0.2736	0.0305	sbp
rs6445583	A	0.2774	0.0349	sbp
rs6452769	A	-0.3143	0.0377	sbp
rs6490019	A	-0.2897	0.0309	sbp
rs6504213	T	-0.2982	0.0312	sbp
rs6539467	A	0.265	0.0404	sbp
rs6562778	A	0.178	0.0304	sbp
rs658780	T	-0.2028	0.0347	sbp
rs665445	A	-0.1909	0.0334	sbp
rs6731373	A	0.1913	0.0326	sbp
rs6737318	A	0.2348	0.0364	sbp
rs6771917	T	-0.3793	0.0355	sbp
rs67885470	T	-0.2087	0.038	sbp
rs6788984	A	0.2999	0.0432	sbp
rs68085857	T	0.274	0.0357	sbp
rs68096471	A	-0.2098	0.0343	sbp
rs68115553	A	-0.6445	0.1143	sbp
rs6870654	T	0.2136	0.0347	sbp
rs6892983	A	0.3427	0.0307	sbp
rs6921291	T	0.3575	0.0385	sbp
rs6957161	A	0.2064	0.0345	sbp
rs6978112	T	0.2286	0.0309	sbp
rs698748	A	0.1871	0.0325	sbp
rs699	A	-0.3748	0.0308	sbp
rs7012866	T	-0.2325	0.0301	sbp
rs702395	T	0.2318	0.0305	sbp
rs7026176	T	-0.1869	0.0299	sbp
rs708117	A	0.2874	0.0302	sbp
rs7093894	A	0.236	0.0427	sbp
rs7107356	A	-0.4598	0.0301	sbp
rs7125196	T	0.4422	0.0472	sbp
rs7134440	T	0.4788	0.0562	sbp
rs7134677	T	-0.3851	0.0332	sbp
rs7154723	A	0.253	0.0309	sbp
rs7186298	T	-0.2315	0.0302	sbp
rs7189884	A	-0.3138	0.0476	sbp
rs7211535	A	-0.1779	0.0304	sbp
rs7213273	A	-0.4	0.0315	sbp
rs7236548	A	0.3431	0.0388	sbp
rs7255933	A	0.2306	0.0345	sbp
rs72683923	T	0.9587	0.1101	sbp
rs72742507	T	-0.2053	0.0328	sbp
rs7278003	T	-0.1876	0.0304	sbp
rs72842207	T	-0.203	0.0367	sbp
rs72847885	A	0.2413	0.0318	sbp
rs73046792	A	-0.3554	0.0426	sbp
rs73049928	A	-0.2382	0.0392	sbp
rs7306710	T	-0.2429	0.0303	sbp
rs73075659	A	0.3962	0.0321	sbp
rs7331680	T	0.4101	0.0423	sbp
rs7338758	T	0.3552	0.0352	sbp
rs73727605	A	0.3616	0.0623	sbp
rs73855810	A	0.2732	0.0434	sbp
rs7395791	A	-0.2162	0.0308	sbp
rs74048190	T	-0.4404	0.0757	sbp
rs743395	T	0.2597	0.0317	sbp
rs7439567	T	0.2537	0.0309	sbp

© 2022 Acosta JN et al. *JAMA Network Open.*

SNP	Effect Allele	Beta	SE	Trait
rs7491248	A	0.2163	0.0362	sbp
rs75016974	T	-0.2513	0.0439	sbp
rs7514579	A	0.2243	0.0361	sbp
rs75461554	T	-0.3016	0.0377	sbp
rs75672964	T	0.5885	0.0839	sbp
rs75961402	A	0.2659	0.0418	sbp
rs7615099	A	0.1891	0.0321	sbp
rs76452347	T	-0.2974	0.0397	sbp
rs76719272	T	-0.2738	0.0461	sbp
rs7683728	T	-0.3654	0.0304	sbp
rs7703560	A	-0.2246	0.0333	sbp
rs7725413	T	-0.1985	0.0359	sbp
rs77375686	A	-0.3467	0.0485	sbp
rs7744902	A	-0.4088	0.0593	sbp
rs7763558	A	0.3363	0.0321	sbp
rs7765526	A	0.201	0.0307	sbp
rs778124	A	0.2965	0.0311	sbp
rs77924615	A	-0.4081	0.039	sbp
rs7821832	T	0.4222	0.0348	sbp
rs7830607	A	-0.206	0.0327	sbp
rs7844887	A	0.2662	0.0363	sbp
rs78474310	A	-0.4699	0.0734	sbp
rs78648104	T	-0.4287	0.0541	sbp
rs786923	T	-0.3082	0.031	sbp
rs79069610	T	-0.4005	0.0727	sbp
rs7912283	A	-0.2144	0.0322	sbp
rs7926110	T	0.2603	0.0321	sbp
rs7926335	T	0.3135	0.0339	sbp
rs7927515	A	0.2271	0.0319	sbp
rs79384779	T	0.3179	0.0428	sbp
rs7944927	T	0.2235	0.0392	sbp
rs79539362	T	0.4003	0.0504	sbp
rs7963801	T	-0.2362	0.0311	sbp
rs79930761	T	-0.4688	0.0559	sbp
rs8003103	A	-0.1755	0.0319	sbp
rs8044992	T	0.2138	0.0331	sbp
rs8054587	T	0.1665	0.0302	sbp
rs8106184	A	-0.2371	0.0347	sbp
rs8125763	A	0.1761	0.0301	sbp
rs8142376	T	0.1676	0.03	sbp
rs8180684	T	0.2134	0.0335	sbp
rs843093	A	-0.2085	0.0338	sbp
rs848445	T	-0.2025	0.0339	sbp
rs869396	A	-0.2115	0.0305	sbp
rs871004	A	0.2336	0.0317	sbp
rs8904	A	0.3061	0.0314	sbp
rs908951	T	-0.2261	0.0315	sbp
rs927315	T	0.1689	0.0303	sbp
rs9302885	A	0.2242	0.0302	sbp
rs9349379	A	0.2664	0.0312	sbp
rs9361836	T	0.2196	0.0324	sbp
rs9368222	A	0.2281	0.0339	sbp
rs9393231	A	-0.2148	0.0309	sbp
rs9401913	A	0.5202	0.0305	sbp
rs9486916	T	0.2657	0.0385	sbp
rs9507885	T	-0.3208	0.0542	sbp
rs9526707	A	-0.2039	0.0323	sbp
rs9549627	A	0.2846	0.05	sbp
rs9857362	A	0.1727	0.0306	sbp
rs9869437	A	-0.2001	0.0318	sbp
rs9876694	T	0.4713	0.0651	sbp
rs9880098	A	0.3081	0.0308	sbp
rs9886665	T	0.2048	0.0343	sbp
rs9897429	A	0.2645	0.0319	sbp
rs9918876	A	-0.2975	0.0498	sbp

© 2022 Acosta JN et al. *JAMA Network Open.*

eTable 3. Results for Individual Life's Simple 7 Components as Factors Associated With Neuroimaging-Related Brain Health Metrics

Life's Simple 7 Score Components	White matter hyperintensities volume		Brain volume	
	Beta (SE)	p	Beta (SE)	p
**Observed (per each category increase)**				
Blood pressure	-0.15 (0.007)	<0.001	0.01 (0.006)	0.06
Cholesterol levels	-0.02 (0.006)	0.003	-0.009 (0.006)	0.11
Glycemic status	-0.12 (0.012)	<0.001	0.10 (0.01)	<0.001
Smoking status	-0.10 (0.008)	<0.001	0.08 (0.007)	<0.001
Physical activity	-0.02 (0.007)	0.004	0.01 (0.006)	0.04
Diet	-0.03 (0.009)	<0.001	-0.02 (0.008)	0.02
Body mass index	-0.13 (0.006)	<0.001	0.05 (0.006)	<0.001
**Genomic (per each category increase)**				
Blood pressure	-0.04 (0.006)	<0.001	-0.008 (0.005)	0.12
Cholesterol levels	-0.01 (0.006)	0.025	0.002 (0.005)	0.76
Glycemic status	-0.007 (0.006)	0.24	-0.001 (0.005)	0.79
Smoking status	-0.02 (0.006)	0.002	0.01 (0.005)	0.04
Physical activity	0.02 (0.006)	<0.001	-0.04 (0.005)	<0.001
Diet	0.001 (0.006)	0.79	-0.004 (0.005)	0.37
Body mass index	-0.03 (0.006)	<0.001	0.02 (0.005)	<0.001

Abbreviations: SE=Standard error.

© 2022 Acosta JN et al. *JAMA Network Open.*

eTable 4. Association Between Life's Simple 7 Scores and Cognitive Measurements in the UK Biobank

Life's Simple 7	Prospective memory		Pairs matching		Fluid intelligence		Reaction time		Symbol digit substitution		Trail making A		Trail making B	
	**OR (95%CI)**	**p**	**Beta (se)**	**p**	**Beta (se)**	**p**	**Beta (se)**	**p**	**Beta (se)**	**p**	**Beta (se)**	**p**	**Beta (se)**	**p**
**Observed**														
**Poor**	Reference		Reference		Reference		Reference		Reference		Reference		Reference	
**Average**	1.19 (1.01-1.39)	0.04	-0.01 (0.02)	0.50	0.22 (0.06)	<0.001	-5.1 (3.1)	0.10	0.82 (0.17)	<0.001	-0.03 (0.02)	0.24	0.002 (0.04)	0.96
**Optimal**	1.19 (1.01-1.40)	0.04	0.01 (0.02)	0.61	0.33 (0.06)	<0001	-6.5 (3.2)	0.04	1.07 (0.18)	<0.001	-0.02 (0.02)	0.36	-0.02 (0.05)	0.62
**Genomic**														
**Poor**	Reference		Reference		Reference		Reference		Reference		Reference		Reference	
**Average**	0.99 (0.91-1.09)	0.88	0.01 (0.01)	0.35	0.04 (0.03)	0.18	-0.75 (1.6)	0.65	0.16 (0.09)	0.09	-0.01 (0.01)	0.34	-0.02 (0.02)	0.46
**Optimal**	1.06 (0.94-1.19)	0.31	0.01 (0.01)	0.46	0.14 (0.04)	0.001	0.61 (2.1)	0.77	0.31 (0.12)	0.008	-0.004 (0.02)	0.78	-0.01 (0.03)	0.66

eTable 4. Prospective memory: Participants were prompted with the following task: "At the end of the games we will show you four coloured shapes and ask you to touch the Blue Square. However, to test your memory, we want you to actually touch the Orange Circle instead." We coded those who completed the task on the first attempt as 1 and the rest as 0. Pairs matching: Participants are asked to memorize the position of as many matching pairs of cards as possible in the fewest tries. Up to three rounds were conducted; we used the second round for our analyses (6 pairs of cards). The final variable comprises the natural log-transformed number of incorrect matches (+1). Fluid intelligence: Participants were tasked with answering as many questions as possible from a pool of 13 verbal logic/reasoning type multiple choice questions within 2 minutes. We used the total number of correct answers (maximum 13) in our analyses. Reaction time: Participants are shown two cards at a time; if both cards are the same, they must press a button as quickly as possible. The test is based on 12 rounds, and, in our analyses, we use the mean time to correctly identify matches. Symbol digit substitution: Participants were presented with one grid linking symbols to single-digit integers, and a second grid containing only the symbols, are asked to indicate numbers attached to each symbol in the second grid using the first one as key. In our analyses, we used the number of correct substitutions, truncating values to the range 1 - 40 to exclude outliers. Trail making A: Participants are tasked with connecting circles containing a sequence of numbers. We use the log-transformed time taken to complete this task (+1) in our analyses. Trail making B: Participants are asked to connect circles containing numbers or letters by alternating between them in ascending sequence. We use the natural log-transformed time taken to complete this task (+1) in our analyses.

© 2022 Acosta JN et al. *JAMA Network Open.*

eTable 5. Stratified Analyses by Sex

	White matter hyperintensities volume				Brain volume			
**Life's Simple 7 Score**	**Female sex**		**Male sex**		**Female sex**		**Male sex**	
	**Beta (SE)**	**p**	**Beta (SE)**	**p**	**Beta (SE)**	**p**	**Beta (SE)**	**p**
**Observed (interaction p value = 0.14)**					**Observed (interaction p value <0.001)**			
**Poor**	Reference		Reference		Reference		Reference	
**Average**	-0.15 (0.038)	<0.001	-0.19 (0.03)	<0.001	0.004 (0.037)	0.91	0.14 (0.03)	<0.001
**Optimal**	-0.32 (0.039)	<0.001	-0.40 (0.04)	<0.001	0.01 (0.04)	0.74	0.23 (0.03)	<0.001
**Genomic (interaction p value = 0.88)**					**Genomic (interaction p value = 0.40)**			
**Poor**	Reference		Reference		Reference		Reference	
**Average**	-0.05 (0.019)	0.008	-0.08 (0.02)	<0.001	0.003 (0.02)	0.87	-0.01 (0.02)	0.48
**Optimal**	-0.08 (0.024)	<0.001	-0.09 (0.03)	0.001	-0.01 (0.02)	0.53	-0.02 (0.02)	0.42

eTable 5. Abbreviations: SE = standard error.

© 2022 Acosta JN et al. *JAMA Network Open.*

eTable 6. Comparison of Performance of Different Models for Each Trait

**Comparison**	
**Model 1**	**Model 2**
WMH volume ~ Observed LS7 (all traits)<br>R^2 = 0.281	WMH volume ~ Observed LS7 (biological)<br>R^2 = 0.276
WMH volume ~ Observed LS7 (all traits)<br>R^2 = 0.281	WMH volume ~ Observed LS7 (lifestyle)<br>R^2 = 0.276
WMH volume ~ Genomic LS7 (all traits)<br>R^2 = 0.269	WMH volume ~ Genomic LS7 (biological)<br>R^2 = 0.269
WMH volume ~ Genomic LS7 (all traits)<br>R^2 = 0.269	WMH volume ~ Genomic LS7 (lifestyle)<br>R^2 =0.269
WMH volume ~ Genomic LS7+Observed LS7<br>R^2 = 0.282	WMH volume ~ Observed LS7<br>R^2 = 0.281
WMH volume ~ Observed LS7<br>R^2 = 0.281	WMH volume ~ Genomic LS7<br>R^2 = 0.269

eTable 6. Abbreviations: WMH = White matter hyperintensities. LS7 = Life's Simple 7. R2 = Coefficient of determination.
*All models include age and sex as covariates.

© 2022 Acosta JN et al. *JAMA Network Open.*

eFigure 1. Development of the Genomic Life's Simple 7 Score

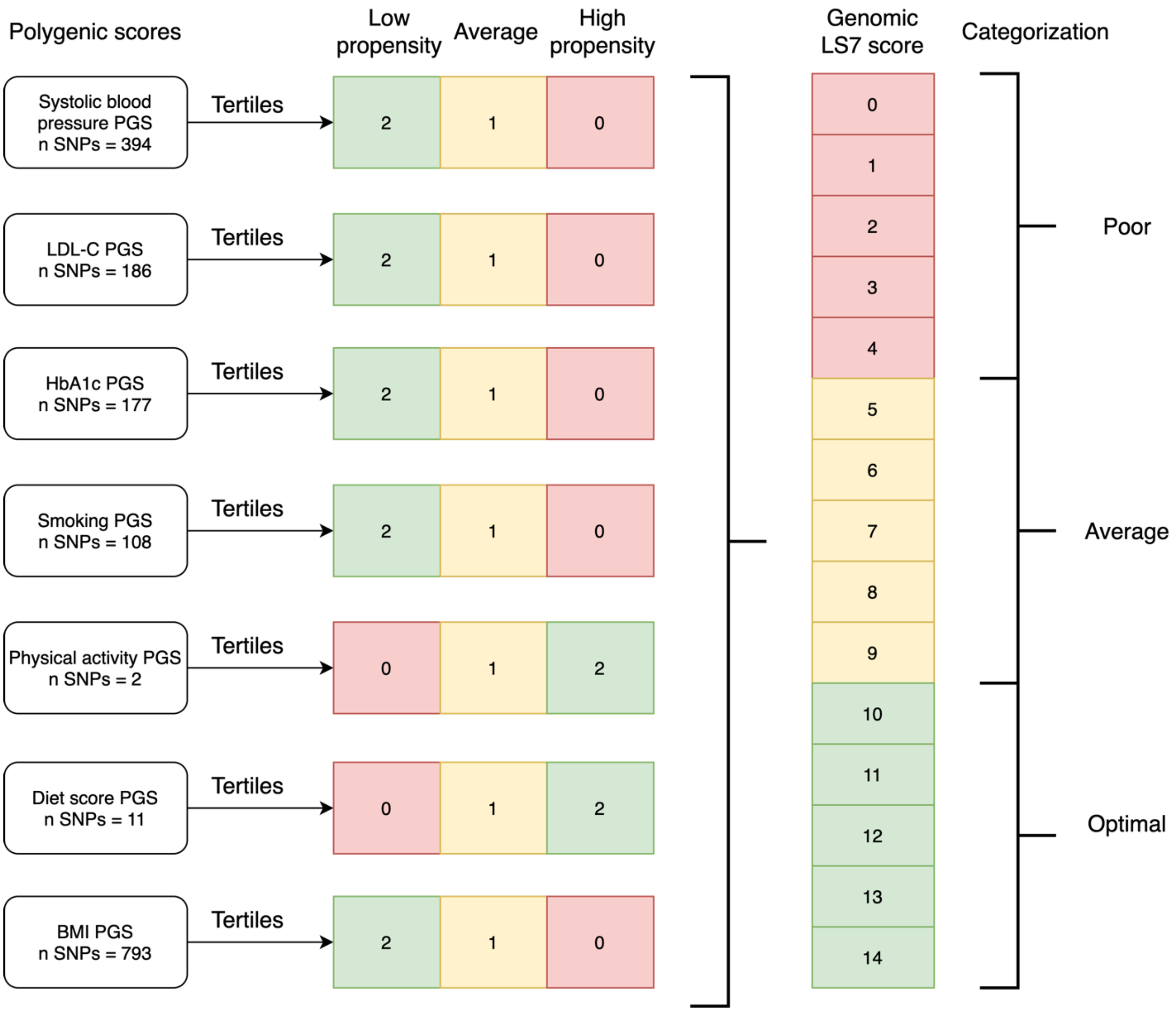

eFigure 1. Each polygenic score representing the genetic propensity for each trait was divided into three groups of equal number of participants, and assigned a value of 0, 1 or 2 depending on the trait. The final genomic LS7 score was the summation among all these categorized polygenic scores. Abbreviations: PGS = Polygenic score. LS7 = Life's Simple 7. SNPs = Single nucleotide polymorphisms. LDL-C = Low density lipoprotein cholesterol. HbA1c = Glycated hemoglobin. BMI = Body mass index.

© 2022 Acosta JN et al. *JAMA Network Open.*

eFigure 2. Flowchart of UK Biobank Participants Included in the Primary Analysis

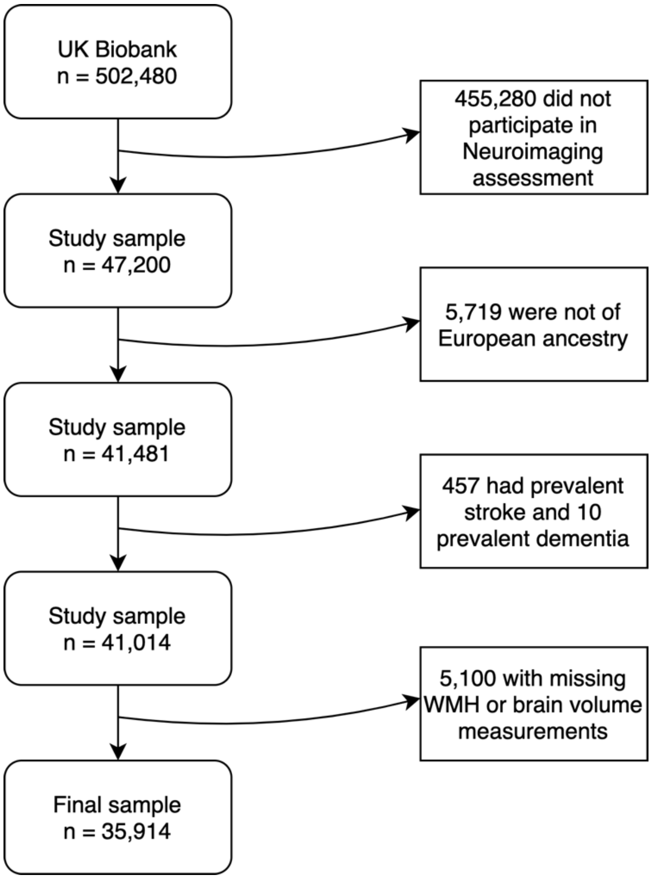

eFigure 2. Abbreviations: UK = United Kingdom. WMH = White matter hyperintensities volume.

© 2022 Acosta JN et al. *JAMA Network Open.*

eFigure 3. Single Nucleotide Variants Included in the Analysis and Overlap Among Traits

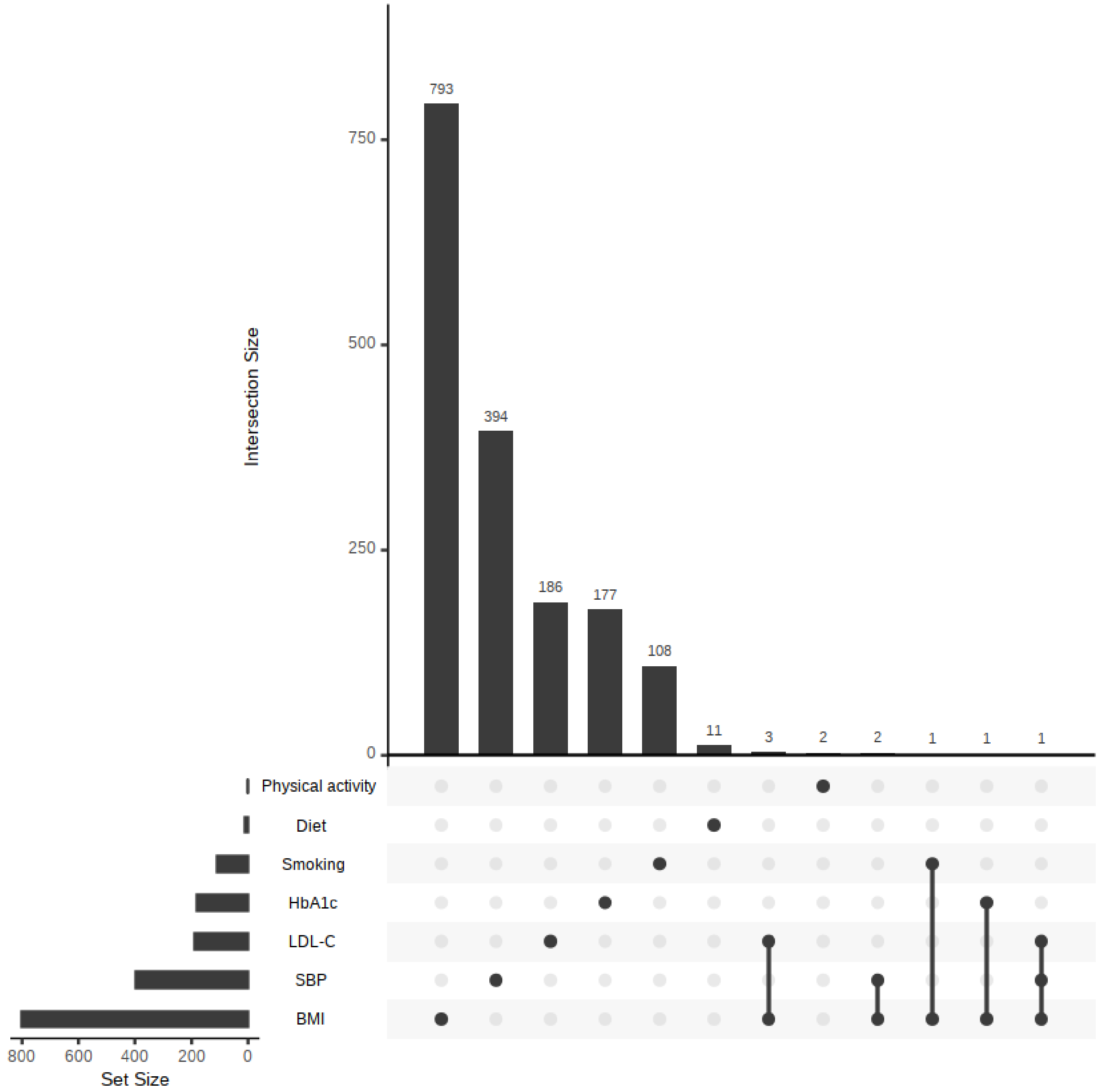

eFigure 3. Abbreviations: HbA1c = Glycated hemoglobin. LDL-C = Low density lipoprotein cholesterol. SBP = Systolic blood pressure. BMI = body mass index.

© 2022 Acosta JN et al. *JAMA Network Open.*
